# Osmophore Structure and Labellum Micromorphology in *Ophrys speculum* (Orchidaceae): New Interpretations of Floral Features and Implications for a Specific Sexually Deceptive Pollination Interaction

**DOI:** 10.3390/plants13101413

**Published:** 2024-05-18

**Authors:** Ana Francisco, Lia Ascensão

**Affiliations:** Centro de Estudos do Ambiente e do Mar (CESAM Lisboa), Faculdade de Ciências da Universidade de Lisboa (FCUL), C2, Campo Grande, 1749-016 Lisboa, Portugal

**Keywords:** *Dasyscolia ciliata* wasp, floral features, flower morphology, labellum micromorphology and anatomy, *Ophrys speculum*, orchid, osmophore, pollination by sexual deception, secretory structure, visual mimicry

## Abstract

Pollination by sexual deception specifically attracts male insects, through the floral scent and particular morphological features of the flower that serve as visual and tactile stimuli. The unique bond between the *Ophrys speculum* orchid and the male *Dasyscolia ciliata* wasp primarily stems from a few distinctive semiochemicals that mimic the female wasp’s sex pheromone, although the floral scent comprises a variety of compounds. An osmophore producing highly volatile compounds has been documented in four close relatives of *O. speculum* and is now being also investigated in this species. Given the existing debates regarding the structure of the labellum and stigmatic cavity in *O. speculum*, this study details their micromorphology. Additionally, comparisons of *O. speculum* flowers and female *D. ciliata* wasps under stereomicroscopy and scanning electron microscopy are conducted to seek new evidence of visual and tactile mimicry. The findings confirm that (i) an osmophore is present at the apical margin of the labellum in *O. speculum* flowers; (ii) the labellum features a distinct basal field homologous to those found in other *Ophrys* species; and (iii) the basal labellum region closely mimics the female wasp’s thorax and wings. The implications of these novel floral features are discussed within an evolutionary context.

## 1. Introduction

Unlike most flowering plants, which reward their pollinators with nectar, pollen, floral oils, liquid fragrances, waxes, or resins, some species evolved a deceptive pollination, offering no real rewards but simulating their presence instead [1,2,3,4,5]. Pollination through deception has been documented across at least 32 angiosperm families, with approximately 87% of these deceptive species found within the family Orchidaceae [3]. An estimated one-third of all orchids, amounting to 6500 to 8000 species, employ deception to attract pollinators [1,3]. Most deceptive orchids are pollinated through generalised food deception, attracting food-foraging insects by mimicking the general signals of rewarding flowers [5,6]. However, a more intricate pollination syndrome, known as sexual deception, has evolved in at least 22 genera of orchids [7,8,9], which deceive only male insects by offering them a set of floral features that mimic their conspecific virgin females [4,10]. Sexual deception typically involves a nearly perfect imitation of the female’s sex pheromone combined with the appearance and texture of the labellum—the modified median petal of the orchid flower [11,12,13,14,15,16,17,18]. Because insect mating signals are species-specific [19], flowers of each sexually deceptive species almost exclusively attract males of one or a few closely related insect species, establishing a highly specialised pollination interaction [11,13,15,17,18,20,21,22,23,24]. Most co-occurring sexually deceptive orchids exhibit strong floral isolation, primarily driven by scent [23,25,26,27,28,29]. Given the weakness or non-existence of postmating barriers, these orchids rely on the robust prepollination reproductive isolation provided by their floral scents and labella to prevent hybridisation [23,24,25,26,28]. Besides floral scent—which has a primary significance for both pollinator attraction and reproductive isolation in sexually deceptive orchids—morphological features of the flowers such as shape, colour, size, and texture provide visual and tactile cues to male pollinators, playing a decisive role in effectively turning attraction into pollination [11,16,18,30,31,32,33,34,35,36,37]. Therefore, characterising the key floral traits involved in the attraction of specific pollinators is crucial to an understanding of the process of pollinator-driven speciation [28,29,36,37,38,39].

The insectiform flowers of the Euro-Mediterranean, sexually deceptive orchid genus *Ophrys* L. (Orchidaceae: Orchidinae) have long fascinated naturalists on account of their distinctive morphology and complex mode of reproduction [40,41,42]. Because of their unique floral scent and special characteristics of their labella, *Ophrys* flowers are visited by mate-searching male insects, predominantly solitary bees but also solitary parasitic wasps, eusocial bees, and certain beetles [7,11,43,44]. While performing copulatory attempts with the labellum—a behaviour known as pseudocopulation—male insects inadvertently remove pollinaria and subsequently serve as pollen vectors on their visits to *Ophrys* flowers [10,45].

The attraction of pollinators to *Ophrys* orchids is initiated by the odour bouquet emitted from the labellum, which comprises over 100 organic compounds with varied volatility and chemical origin [46,47]. This bouquet includes a small group of key semiochemicals that effectively trigger mating behaviour in specific male insects, due to their resemblance to the female’s sex pheromones [12,17,24,48,49,50,51].

The highly specific one-to-one pollination relationship between *O. speculum* Link and the solitary parasitic wasp *Dasyscolia ciliata* (Hymenoptera: Scoliidae) was first reported in the early twentieth century by Pouyanne, marking a pivotal discovery in the study of pollination by sexual deception [10,41]. The specificity of this relationship largely stems from the unique chemical mating signal emitted by the wasp, which the orchid mimics. This signal consists in a few polar, short-chained organic compounds—specifically aldehydes, ethyl esters, and three rare oxygenated carboxylic acids, two of which are in specific enantiomeric ratios [38,48]. However, the precise areas of the labellum responsible for the biosynthesis of these compounds remain unidentified and the odour bouquet of *O. speculum* includes a broad range of other volatile organic compounds [38].

It is likely that at least some components of the *Ophrys* floral scent, particularly those with higher volatility, are synthesised in a secretory structure known as an osmophore, located in the apical region of the labellum [52]. The presence of this fragrance-producing gland has been confirmed in four closely related *Ophrys* species: *Ophrys bombyliflora* Link, *O. fusca* Link, *O. lutea* Cav., and *O. tenthredinifera* Willd. [52,53,54]. Molecular phylogenetic analyses within the genus *Ophrys* have shown that *O. bombyliflora*, *O. tenthredinifera*, and section *Pseudophrys* Godfery (which includes *O. fusca* and *O. lutea*) together form a major clade (i.e., a monophyletic group) with *O. speculum*, although the interspecific relationships in this clade are not fully resolved yet [55,56,57,58]. Therefore, determining whether the labellum of *O. speculum* also contains an osmophore as its closest relatives and examining its floral features are fundamental to find potentially informative micromorphological data that could aid not only in clarifying the phylogenetic relationships but also in reconstructing the ancestral character states within this clade [59].

Furthermore, the pronounced visual resemblance between the flower of *O. speculum* and the female of its pollinator underlines the significant role that visual signals should play in facilitating successful pseudocopulation by male insects [11,43,60]. Indeed, micromorphological studies of the labellum in various *Ophrys* species, along with comparisons to the females of their pollinators, have identified characteristics indicative of visual and tactile mimicry [47,52,54,60,61,62,63,64,65]. Although the general micromorphology of the labellum in *O. speculum* is well documented [60,64,65,66], a comprehensive description of this flower’s structure is crucial due to existing debates over the configuration of the stigmatic cavity and the presence of a distinct basal field in the labellum of *O. speculum* [59,64,66,67,68].

The concept of basal field was initially defined, and used thereafter, as a distinct area in the basal region of the labellum of *Ophrys* flowers, located above the speculum and below the stigmatic cavity [66,68]. This area typically features a colouration that either contrasts with or is similar to adjacent areas. In most *Ophrys* species, this differentiated area is readily apparent, yet it is absent in species from the section *Pseudophrys* [59,66,68]. The presence of a basal field in the labellum of *O. speculum*, however, has sparked debate as various authors have applied the term “basal field” to different floral regions. Some suggest that in this species, the labellum only displays a poorly defined, glabrous basal field due to its flat epidermal cells [64,67]. Yet, these cells closely resemble those typically found not in the labellum but in the contiguous floor of the stigmatic cavity of other *Ophrys* species [52,59], casting doubt on their homology. Accurately interpreting the floral structure of *O. speculum* requires a detailed micromorphological comparison with other *Ophrys* species to establish homology between floral parts.

Building on our previous studies of the labellum micromorphology and anatomy within the clade composed of the groups of *O. bombyliflora*, *O. tenthredinifera*, *O. speculum,* and section *Pseudophrys* [52,54], the present study focuses on the two subspecies of *O. speculum* that occur in the western part of its geographic range: *O. speculum* Link subsp. *lusitanica* O.Danesch & E.Danesch and *O. speculum* Link subsp. *speculum*. The objectives of this study are the following: (i) to investigate the presence of an osmophore in *O. speculum*, detailing its structure, location, and secretion; (ii) to characterise the micromorphology of the labellum and stigmatic cavity, aiming to establish homologies between floral parts that may resolve the ongoing debate regarding the presence of a distinct basal field in the labellum of this species; and (iii) to conduct a micromorphological comparison between the flower of *O. speculum* and the female of its specific wasp pollinator, seeking structural similarities that could provide new evidence of visual and tactile mimicry.

## 2. Results

### 2.1. Flower Morphology

**Figure 1:**
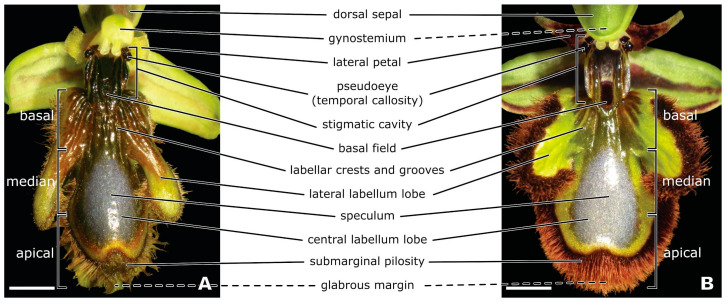
Macrographs of flowers of *Ophrys speculum* subsp. *lusitanica* (**A**) and *Ophrys speculum* subsp. *speculum* (**B**) showing the principal morphological floral features. The stigmatic cavity and the three main regions of the labellum are delimited. The concealed glabrous margin of the labellum is indicated by dashed lines. Scale bars = 2.5 mm.

**Figure 2:**
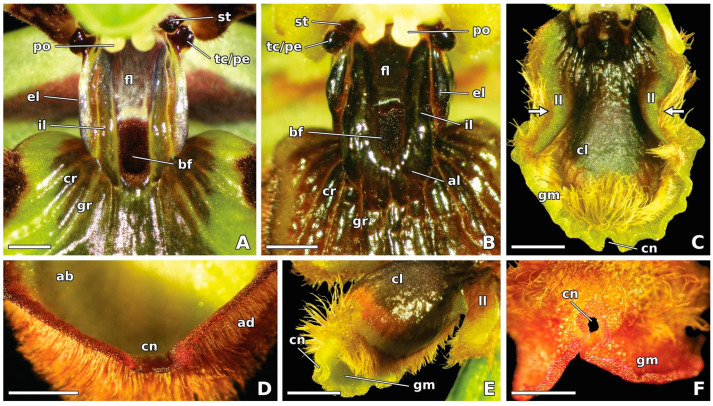
Stereomicrographs of fresh flowers of *Ophrys speculum* subsp. *speculum* (**A**,**D**) and *Ophrys speculum* subsp. *lusitanica* (**B**,**C**,**E**,**F**). (**A**,**B**) Details of the stigmatic cavity and the contiguous basal region of the labellum of open flowers, showing their main components. (**C**) Late bud just before the anthesis with the labellum not fully expanded. Note the natural yellow pigmentation of the glabrous apical margin of the labellum and the knee-shaped bend in the lateral labellum lobes (arrows). (**D**) Open flower (in back view), showing the natural red pigmentation of the glabrous apical labellum margin with a central notch. (**E**) Open flower (in lateral view), showing the convex central labellum lobe. Note the glabrous margin folded at the central notch. (**F**) Freshly opened flower (in back view) after immersion in diluted neutral red, showing the red-stained apical labellum margin. Scale bars: (**A**,**B**) = 1 mm; (**D**,**F**) = 2 mm; (**C**,**E**) = 2.5 mm. Abbreviations: ab = abaxial surface; ad = adaxial surface; al = augmented portion of labellum; bf = basal field; cl = central labellum lobe; cn = central notch; cr = labellar crest; el = external labium; fl = floor of stigmatic cavity; gm = glabrous margin; gr = labellar groove; il = internal labium; ll = lateral labellum lobe; pe = pseudoeye; po = pollinarium; st = staminodial callosity; tc = temporal callosity.

The distinctive flowers of *O. speculum* are easily recognised by their convex, obovate, trilobed labellum which features a wide, shiny, blue speculum bordered by an orange-to-green band and a dense, long submarginal pilosity that conceals a broad, undulated, glabrous apical margin with a central notch (Figure 1 and Figure 2C–E). In *O. speculum* subsp. *lusitanica*, the pronounced convexity of the central labellum lobe often causes the glabrous apical margin to fold longitudinally at the central notch, resulting in the two halves meeting (Figure 2C,E). The two lateral lobes of the labellum are convex with the tip bending backward due to a knee-shaped bend in the middle in *O. speculum* subsp. *lusitanica* (Figure 1A and Figure 2C,E), whereas in *O. speculum* subsp. *speculum*, they are flat with the tip pointing forward (Figure 1B).

The stigmatic cavity of *O. speculum* flowers features an extensive, elevated, flat floor that is bordered by two long, protruding labia or crests—one internal and another external—on each side (Figure 2A,B). These labia are considered to be forward extensions of the lower portion of the walls of the stigmatic cavity flanking its floor (Figure 2A,B). A pair of shiny, black pseudoeyes emerges from the temporal callosities, situated in the upper portion of the stigmatic cavity, directly beneath a pair of black staminodial callosities. The sticky stigmatic surface is located at the ceiling of this cavity, below the pollinaria (Figure 2A,B).

The prominent stigmatic cavity of *O. speculum* seems to join the basal region of the labellum in an unusual manner. A confined basal field—a part of the labellum—appears elevated and restricted to a cupuliform (cup-shaped) concavity, which is located between the distal portions of the internal labia of the stigmatic cavity and is surrounded, on its distal side, by an augmented portion of the labellum fused with these labia (Figure 2A,B). The area of the labellum directly beneath it, which is placed in a lower level, exhibits a sulcate texture due to a series of grooves and crests that diverge radially from the constricted area between the stigmatic cavity and the basal region of the labellum (Figure 1 and Figure 2A,B).

### 2.2. Micromorphology of the Labellum and Stigmatic Cavity

**Figure 3:**
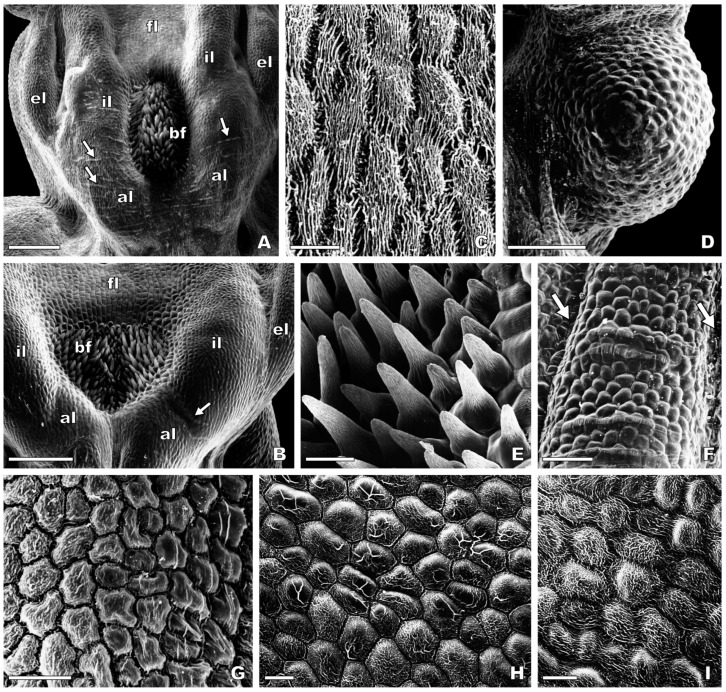
Scanning electron micrographs of the adaxial surface of the stigmatic cavity and the basal region of the labellum in flowers at anthesis of *Ophrys speculum* subsp. *lusitanica* (**A**,**D**,**F**) and *Ophrys speculum* subsp. *speculum* (**B**,**C**,**E**,**G**–**I**). (**A**,**B**) Basal region of the labellum contiguous to the stigmatic cavity. The basal field is confined to a cupuliform concavity between the two internal labia of the stigmatic cavity, which are fused distally with an augmented portion of the labellum, where rows of ordered cuticular ridges are evident (arrows). (**C**) Densely striated flat epidermal cells in the floor of the stigmatic cavity (cell type 1). (**D**) Smooth lenticular cells in the pseudoeye (cell type 2). (**E**) Sub-conical trichomes in the basal field near the periphery of the cupuliform concavity (cell type 3). (**F**) Labellar crest flanked by grooves (arrows). (**G**) Detail of a labellar crest showing the transition between densely striated cells (towards the groove) (cell type 4) and nearly smooth cells (towards the top of the crest). (**H**,**I**) Lateral labellum lobe. (**H**) Apical area, showing lenticular cells with a polygonal outline exhibiting either a reticulate or a heterogeneous cuticular pattern on their surface (cell type 4). (**I**) Basal area, near the transition to the central labellum lobe. Note the diffuse arrangement of the cuticular striations. Scale bars: (**A**,**B**) = 500 μm; (**D**) = 250 μm; (**E**,**F**) = 100 μm; (**C**,**G**–**I**) = 30 μm. Abbreviations: al = augmented portion of labellum; bf = basal field; el = external labium; fl = floor of stigmatic cavity; il = internal labium.

**Figure 4:**
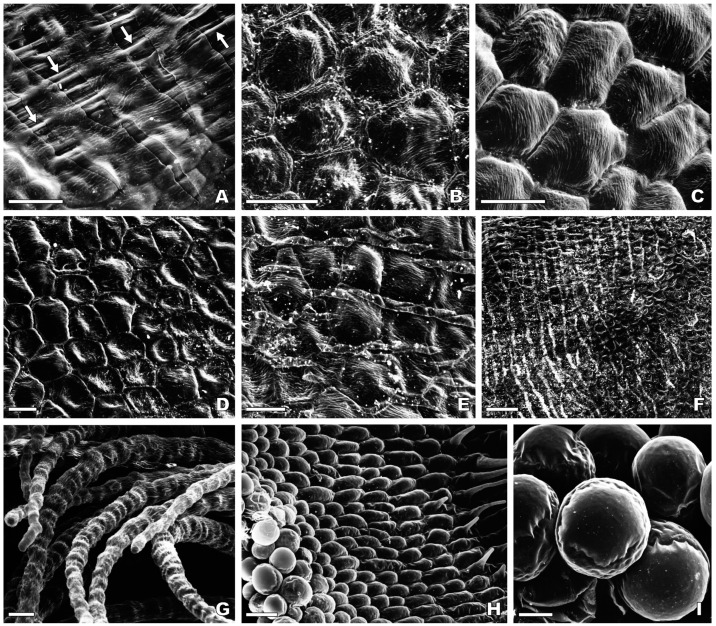
Scanning electron micrographs of the adaxial surface of the median and apical regions of the labellum in flowers at anthesis of *Ophrys speculum* subsp. *speculum* (**A**,**B**,**G**–**I**) and *Ophrys speculum* subsp. *lusitanica* (**C**–**F**). (**A**) Detail of the basal-median region of the labellum, near the speculum. A thin film covering the cell surface seems to have been broken into several strips, revealing long and dense cuticular depositions underneath (arrows). (**B**,**C**) Central area of the speculum, showing flat to lenticular cells with a hexagonal, pentagonal, or heptagonal outline (cell type 6). Note the fine, parallel-arranged cuticular striations on their surface. (**D**–**F**) Apical area of the speculum. (**D**) Flat epidermal cells coexist with cells with a central depression. (**E**) Speculum cells with strips of cuticular depositions, near the labellum margin. (**F**) General view of the rows of cuticular depositions that are laid over some cells near the labellum margin. (**G**) Long contorted trichomes of the submarginal band in the apical labellum (cell type 5). (**H**) Glabrous apical margin of the labellum. (**I**) Detail of dome-shaped papillae of the apical labellum margin (cell type 7). Scale bars: (**A**–**E**,**G**,**I**) = 30 μm; (**F**,**H**) = 100 μm.

Scanning electron microscopic observations revealed that the flowers of *O. speculum* exhibit seven morphologically distinct epidermal cell types based on cell surface’ features in the adaxial surface of the labellum and stigmatic cavity (Figure 3 and Figure 4). The abaxial labellum surface is composed of smooth pavement cells, except for the apical margin, where large dome-shaped papillae occur.

#### 2.2.1. Stigmatic Cavity

The floor of the stigmatic cavity of *O. speculum* is composed of flat epidermal cells with elongated, polygonal outline and dense parallel-arranged cuticular striations on their surface (cell type 1) (Figure 3C). Apart from the floor, the stigmatic cavity exhibits basically smooth, flat to lenticular cells with an isodiametric, polygonal outline (cell type 2), particularly in the pseudoeyes (temporal callosities), staminodial callosities, internal labia, and external labia (Figure 3A,B,D). Prominent cuticular ridges were found over the surface of some epidermal cells, forming perpendicular rows in the internal labia, the augmented portion of the labellum contiguous to the basal field and labellar crests and grooves (Figure 3A,B,F).

#### 2.2.2. Basal Region of the Labellum

The basal field in *O. speculum* consists of sub-conical to attenuate unicellular trichomes with fine cuticular striations on their surface (cell type 3), which are restricted to the cupuliform concavity occurring in the area of confluence between the labellum and the stigmatic cavity (Figure 3A,B,E). The sulcate area of labellar crests and grooves, which extends into the lateral labellum lobes (Figure 1), is composed of either cells similar to those occurring in the internal labia in *O. speculum* subsp. *lusitanica* (cell type 2) (Figure 3F), or lenticular cells with an isodiametric, polygonal outline and a rugose surface (cell type 4), which acquire a nearly smooth appearance towards the top of the crests in *O. speculum* subsp. *speculum* (Figure 3G).

#### 2.2.3. Lateral Labellum Lobes

The lateral lobes of the labellum in *O. speculum* present a wide glabrous surface surrounded by a dense marginal indumentum of long contorted unicellular trichomes (cell type 5), similar to those occurring in the sub-marginal indumentum of the central labellum lobe (Figure 4G). The glabrous portion of the lateral lobes is usually composed of lenticular cells with either a smooth or slightly striated surface (cell type 2) in *O. speculum* subsp. *lusitanica* or a densely striated surface (cell type 4) in *O. speculum* subsp. *speculum*, in which the cuticular ornamentation varies according to the area of the lateral lobe (Figure 3H,I). In the apical area of the lobe, a heterogeneous cuticular pattern consisting of irregular and prominent ridges associated with smooth zones was often found in the surface of some cells (Figure 3H).

#### 2.2.4. Central Labellum Lobe

The major portion of the central labellum lobe in *O. speculum* is occupied with the speculum, which is composed of flat to slightly convex epidermal cells with a hexagonal outline and very fine, parallel cuticular striations on their outer cell wall (cell type 6) (Figure 4B,C). Lenticular cells with flattened anticlinal fields and cells with a pentagonal or heptagonal outline were occasionally found in the speculum amongst the most common cells with a hexagonal outline (Figure 4B,C). In the apical area of the speculum, several epidermal cells exhibit a central depression on their surface (Figure 4D) and long, prominent cuticular depositions were found to be laid over some contiguous cells (Figure 4E). Rows of these ordered, parallel-arranged cuticular depositions are particularly extensive near the apical margin in *O. speculum* subsp. *lusitanica* (Figure 4F) and in the basal area of the speculum. A thin film seems to cover the labellum surface in *O. speculum* subsp. *speculum* and appears to have been split into several strips in the basal area of the speculum near the labellar grooves and crests, revealing the long and dense cuticular depositions that occur underneath (Figure 4A). The speculum is surrounded by a submarginal band of long contorted trichomes (cell type 5) (Figure 4G) and then by a broad, glabrous apical margin composed of smooth, dome-shaped papillae, which become highly voluminous towards the labellum margin itself (cell type 7) (Figure 4H,I).

### 2.3. Micromorphology of the Female Wasp

**Figure 5:**
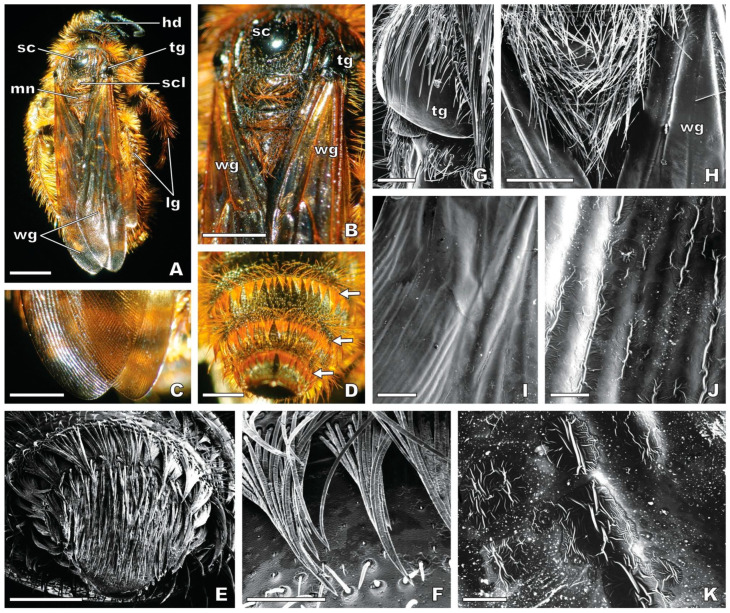
Female *Dasyscolia ciliata* subsp. *ciliata* wasp. (**A**–**D**) Stereomicrographs. (**A**) Dorsal surface of the wasp. (**B**) Detail of the dorsal surface of the thorax, showing a hairy triangular area corresponding to the visible portion of the scutellum and the metanotum, which is delimited by the two glabrous forewings. The glabrous scutum and tegulae are also evident. (**C**) Detail of the apex of the crossed forewings forming an inverted V—note their rippled texture. (**D**) Ventral surface of the abdomen. A band of hair tufts is clearly visible on each segment (arrows). (**E**–**K**) Scanning electron micrographs. (**E**) Bottom view of the abdomen, showing its last segment and its apical bands of hairs. (**F**) Detail of the hairs grouped in tufts on the abdomen dorsal surface. (**G**) Glabrous tegula. (**H**) Uncovered portion of the hairy metanotum delimited by the two glabrous forewings. (**I**) Forewing. Note the absence of hairs on its rippled surface. (**J**) Detail of the forewing dorsal surface, showing parallel arranged cuticular wrinkles. (**K**) Fine cuticular pattern of the forewing. Scale bars: (**A**) = 3 mm; (**B**–**D**) = 2 mm; (**E**,**H**) = 1 mm; (**I**) = 500 μm; (**F**,**G**) = 250 μm; (**J**,**K**) = 100 μm. Abbreviations: hd = head; lg = leg; mn = metanotum; sc = scutum; scl = scutellum; tg = tegula; wg = forewing.

Similar morphological features have been found in the female *Dasyscolia ciliata* subsp. *ciliata* wasp captured in Serra de Sicó for this study (Figure 5A–D) and in the three examined specimens of this wasp from Museum collections. The body and the legs of the female wasp are mostly covered by a brownish-orange-coloured pilosity (Figure 5A) which consists of long, thin, straight hairs (Figure 5B,H). The abdominal segments exhibit slightly spiralled hairs grouped in tufts that are arranged into a band around each segment (Figure 5D–F).

The two pairs of wings are basically glabrous (Figure 5H,I). The two crossed forewings, which partially overlap each other, form a small inverted V at the apex (Figure 5A,C) and are provided with several conspicuous veins, particularly at the proximal area (Figure 5B). The prominent costal veins in the proximal area of the crossed forewings (Figure 5H) compare roughly with the two pairs of labia of the stigmatic cavity of the flower (Figure 3A,B). The veins of the median area of the wings (Figure 5B) are reminiscent of the series of labellar grooves and crests (Figure 2A,B). The forewings’ surface is rippled due to a series of parallel, longitudinal striations (Figure 5C,I) which are frequently associated with cuticular folds and irregular wrinkles (Figure 5J,K). The rippled surface of the wings’ distal area (Figure 5C,I) compares with the rows of ordered cuticular depositions that appeared parallel to the apical margin of the labellum (Figure 4F). Also, the cuticular wrinkles of the wings (Figure 5J,K) resemble the heterogeneous cuticular pattern of the lateral labellum lobes (Figure 3H).

A glabrous scale-like sclerite, the tegula, covers the base of the wings near the point of attachment to the thorax (Figure 5A,B,G). The two tegulae have resemblance with the pseudoeyes of the flower (Figure 3D). Two distinct areas are visible in the dorsal region of the mesothorax: (i) the scutum, a glabrous black-shining area; and (ii) the scutellum, a smaller hairy area that presents a pilosity similar to the region occurring immediately below, the metanotum (Figure 5A,B,H). The glabrous scutum of the wasp (Figure 5B) compares well with the glabrous floor of the stigmatic cavity of the orchid flower (Figure 2A,B). A hairy triangle is visible between the proximal areas of the crossed forewings, showing the hairiness of the portions of the scutellum and the metanotum not hidden by the wings (Figure 5B,H). This triangular-shaped area roughly compares to the oblong to shield-shaped basal field confined to the cupuliform concavity of the labellum (Figure 2A,B and Figure 3A,B).

### 2.4. Location, Structure, and Secretion of the Osmophore

**Figure 6:**
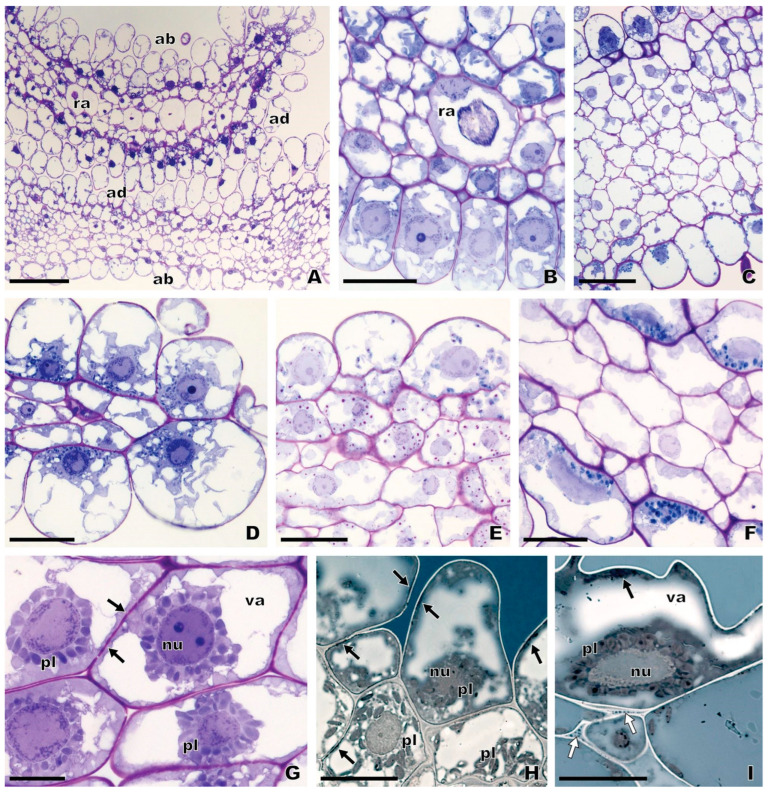
Light micrographs of sections of the apical region of the labellum in *Ophrys speculum* subsp. *lusitanica* (**A**,**C**,**F**–**I**) and *Ophrys speculum* subsp. *speculum* (**B**,**D**,**E**). (**A**–**G**) Historesin sections stained with toluidine blue/Lugol (**A**,**G**) or PAS/toluidine blue (**B**–**F**). (**A**,**B**,**D**,**E**) Transverse sections of the glabrous labellum margin. (**A**) Early bud, showing two different portions of the labellum margin. Note that the most apical portion (upper half) presents epidermal cells with a denser cytoplasm in both abaxial and adaxial surfaces. (**B**) Early bud, showing secretory epidermal cells on the abaxial surface and a raphide-containing idioblast in the parenchyma. (**D**) Detail of secretory dome-shaped papillae of the labellum margin in a late bud. (**E**) Early bud, showing PAS-positive, pink-stained plastids with low starch content in the subepidermal parenchyma cells on the abaxial surface. (**C**) Transverse section of the labellum area contiguous to the glabrous margin in a late bud, showing highly vacuolated epidermal cells on the abaxial surface. (**F**) Longitudinal section of the glabrous labellum margin in an open flower, showing no starch in the highly vacuolated parenchyma cells. (**G**) Paradermal section of epidermal cells in a late bud. Note the primary pit fields (arrows) along cell walls and the perinuclear arrangement of plastids. (**H**,**I**) Transverse epoxy resin sections of the secretory tissues of the labellum margin in an early bud (**H**) and an open flower (**I**) stained with Sudan Black B. A black-staining lipophilic secretion is visible mainly near the outer epidermal cell walls (black arrows) and in the loose inner cell walls (white arrows). Scale bars: (**A**) = 150 μm; (**C**) = 100 μm; (**B**,**D**–**F**) = 50 μm; (**G**–**I**) = 25 μm. Abbreviations: ab = abaxial surface; ad = adaxial surface; nu = nucleus; pl = plastids; ra = raphides; va = vacuole.

**Figure 7:**
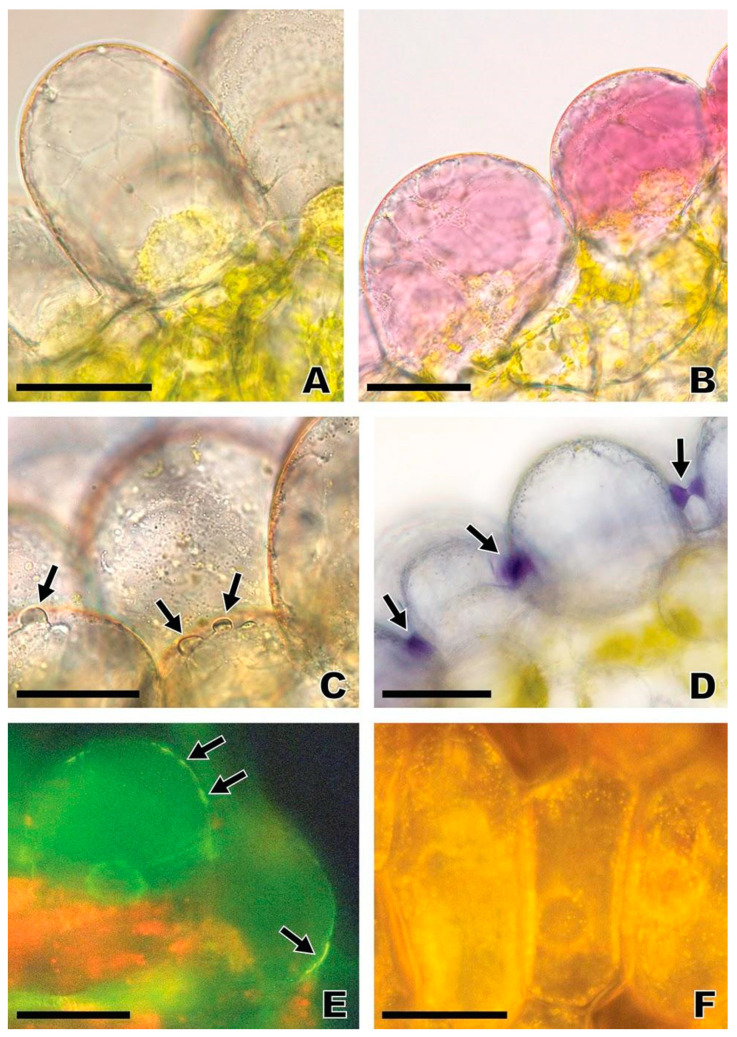
Histochemical characterisation of fresh hand-cut sections of the apical margin of the labellum in *Ophrys speculum* subsp. *lusitanica* (**A**,**C**–**E**) and *Ophrys speculum* subsp. *speculum* (**B**,**F**). (**A**–**C**) Transverse sections without any treatment (controls). (**A**) Late bud just before the anthesis, showing dome-shaped papillae with a clear polarity. (**B**) Open flower, showing the natural magenta pigmentation of the anthocyanin-rich vacuoles in the epidermal cells. (**C**) Translucent droplets (arrows) on the surface of the epidermal cells in an open flower. (**D**,**E**) Transverse sections of open flowers. (**D**) Violet-stained droplets of secretion (arrows) are visible on the surface of epidermal cells after staining with Nadi reagent. (**E**) Autofluorescence under blue light. Note a bright-green autofluorescence near the outer epidermal cell walls (arrows) and the typical red autofluorescence of the chloroplasts in the parenchyma cells. (**F**) Paradermal section of an open flower stained with neutral red. Lipophilic inclusions show a golden-yellow secondary fluorescence under blue light. Scale bars = 50 μm.

Flowers of the two studied subspecies of *O. speculum* emanate a faint-smelling scent and exhibit a period of anthesis that lasts 12–17 d (A. Francisco, personal observation). The glabrous apical margin of the labellum of freshly opened flowers of *O. speculum* subsp. *lusitanica* stained an intense red rapidly after immersion in diluted neutral red (Figure 2F). Flowers of *O. speculum* subsp. *speculum* were not tested with this stain because of the natural magenta-to-red pigmentation of the epidermal cells occurring in the apical labellum margin (Figure 2D), which would interfere with the result of the test. The quick absorption of the diluted neutral red by the dome-shaped papillae of the apical margin provided a first indication of their secretory nature, which was subsequently confirmed by the results of the anatomical and histochemical study (Figure 6 and Figure 7).

**Figure 8:**
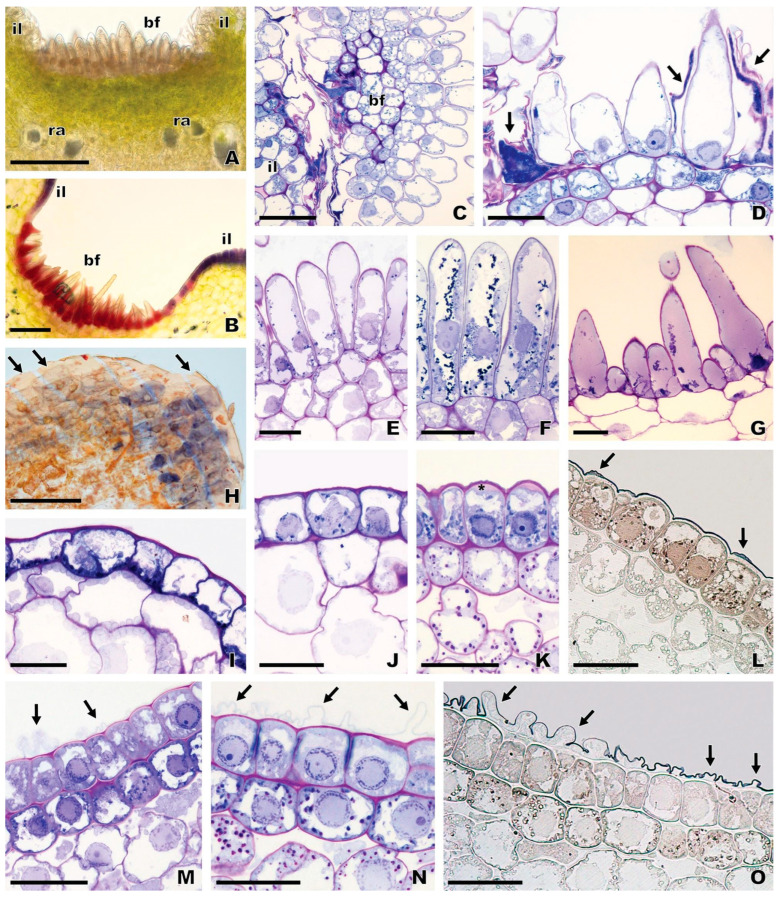
Light micrographs of sections of the basal and median regions of the labellum in *Ophrys speculum* subsp. *lusitanica* (**A**,**C**–**E**,**I**,**K**,**L**) and *Ophrys speculum* subsp. *speculum* (**B**,**F**–**H**,**J**,**M**–**O**). (**A**–**G**) Basal field of the labellum. (**A**,**B**) Transverse fresh hand-cut sections of a late bud stained with Sudan IV (**A**) and an open flower without any treatment (**B**). Sub-conical to attenuate trichomes are observed in the basal field located in the cupuliform concavity. (**C**–**G**) Historesin sections stained with PAS/toluidine blue (**C**,**D**,**F**,**G**) or toluidine blue/Lugol (**E**). (**C**,**D**) Paradermal sections of the basal field in an early bud, showing trichomes in their transverse extent in D. Note the intense dark-blue-stained content of some collapsed cells (arrows). (**E**–**G**) Sections of the densely packed trichomes of the basal field in late buds (**E**,**F**) and an open flower (**G**). Note the dark-greenish-blue-stained granular vacuolar inclusions. (**E**,**G**) Transverse sections. (**F**) Longitudinal section. (**H**,**I**) Labellar crests near the basal area of the speculum in open flowers. (**H**) Oblique fresh hand-cut section stained with Sudan IV. A red-stained lipophilic film split into several strips (arrows) seems to cover the labellar crest surface. (**I**) Transverse historesin section stained with PAS/toluidine blue, showing highly vacuolated epidermal and parenchyma cells. (**J**–**O**) Historesin sections of the speculum stained with PAS/toluidine blue (**J**,**K**,**M**,**N**) or Sudan Black B (**L**,**O**). (**J**) Central-median area of the speculum in a late bud, showing epidermal cells with a nearly smooth surface. (**K**,**L**) Lateral-median area of the speculum in an early bud, showing epidermal cells with a slightly ridged surface and some cells with a distended cuticle forming small subcuticular spaces where a Sudan-positive lipophilic material is seen (arrows). A pink-stained material is also observed in the periplasmic space of some epidermal cells (asterisk). (**M**–**O**) Lateral-apical area of the speculum in an early bud. Irregular cuticular projections with an underlying subcuticular space (arrows) are well seen. Scale bars: (**A**,**B**) = 150 μm; (**C**,**H**) = 100 μm; (**D**–**G**,**I**–**O**) = 50 μm. Abbreviations: bf = basal field; il = internal labium; ra = raphides.

Indeed, contrary to the other regions of the labellum, these dome-shaped papillae present typical features of secretory cells, namely a dense cytoplasm, a large nucleus lying at the base of the cell, numerous plastids, several small vacuoles at the cell apex, and thin cell walls (Figure 6 and Figure 7A). Primary pit fields were also found between epidermal cells (Figure 6G). The pleiomorphic plastids in the domed papillae exhibit typical perinuclear distribution (Figure 6G–I and Figure 7A,E) and lack chlorophyll pigments, since they did not show their characteristic red autofluorescence under blue light (Figure 7E). Conversely, large fusiform to oblong chloroplasts abound in the subepidermal parenchyma cells of the apical labellum margin (Figure 6H and Figure 7A,B,E). A few starch grains were found in these plastids only at the earliest stage of the flower development (Figure 6E,F). Crystal idioblasts containing raphides are frequent in the parenchyma tissue of the glabrous margin, basal field, and speculum, especially in early buds (Figure 6A,B and Figure 8A). In contrast to the other labellum areas, which exhibit highly vacuolated epidermal cells at the stages of late bud and open flower (Figure 6C and Figure 8G,I,J), the cells at the glabrous apical margin maintain their secretory characteristics, such as dense cytoplasm, throughout the flower’s development, even though an increase in cell vacuolisation had occurred from early buds to flowers at anthesis (Figure 6B,E,F,H,I). These histological features indicate that an osmophore occurs in the glabrous apical margin, which appears restricted to the area nearest the margin itself, where the labellum thickness reaches a maximum of three-to-four layers of parenchyma cells (Figure 6A,D,F). This finding is consistent with the most intense staining that this area exhibited after immersion in diluted neutral red (Figure 2F). The osmophore of *O. speculum* is thus composed of the dome-shaped papillae, together with the underlying two-to-three layers of parenchyma cells, which occur in both the abaxial and the adaxial surfaces of the apical region of the labellum nearest the margin.

The histochemical tests revealed a low amount of a lipophilic secretion associated with the domed papillae at the apical labellum margin of *O. speculum* (Figure 6H,I and Figure 7C–F). The translucent droplets that were often observed on the surface of the domed papillae in fresh material (Figure 7C) stained violet with the Nadi reagent (Figure 7D), which indicates the presence of terpenoids with low molecular mass. A Sudan-positive lipophilic secretion was observed in close association with the epidermal cell walls, most likely in the periplasmic space and in the loose inner cell walls (Figure 6H,I), and some lipophilic inclusions were also detected in the cortical cytoplasm of the domed papillae (Figure 7F). In addition, an autofluorescence in blue (under UV light; not shown) that changed to an autofluorescence in bright green (under blue light), which are assigned to phenolic compounds, were detected near the outer epidermal cell walls (Figure 7E). 

### 2.5. Anatomy of the Basal Field and Speculum

The distinctive basal field of *O. speculum* is composed of densely packed trichomes located in a cupuliform concavity (Figure 8A,B), which appeared oblong in transverse and longitudinal sections and exhibited granular phenolic inclusions in their vacuoles (Figure 8E–G). In some early buds of *O. speculum* subsp. *lusitanica*, certain trichomes were found to be collapsed, a finding that was shown by the intense dark blue colour of their protoplasm in sections stained with toluidine blue (Figure 8C,D). However, we did not find any indication of collapsed cells at later stages of flower development in *O. speculum* subsp. *lusitanica* (Figure 8E) nor did we at early buds, late buds, or flowers at anthesis of *O. speculum* subsp. *speculum* (Figure 8F,G).

The epidermis of the speculum consists of cells that appeared quadrangular to rectangular in the transverse section, which exhibited a nearly smooth cuticle at the stages of late bud and open flower (Figure 8J), and a slightly ridged cuticle (Figure 8K,L), which occasionally acquired an uncommon configuration, in the lateral area of the speculum at the early bud stage (Figure 8M–O). Indeed, the portion of the speculum that is folded over itself in an early bud of *O. speculum* subsp. *speculum* displayed a series of contiguous epidermal cells whose cuticle had been detached from their outer tangential walls, in such a way that irregular cuticular projections had been formed with an underlying subcuticular space (Figure 8M–O). Apparently, the content of this subcuticular space did not stain with any staining procedure used here. Conversely, in *O. speculum* subsp. *lusitanica*, the cuticle of some speculum cells was found to be distended at some places, forming small subcuticular spaces where a Sudan-positive lipophilic material seemed to accumulate (Figure 8L). Also, some of these epidermal cells exhibited a conspicuous periplasmic space which appeared pink after staining with PAS reagent plus toluidine blue (Figure 8K).

## 3. Discussion

### 3.1. The Basal Field of Ophrys speculum

By definition, the basal field in *Ophrys* flowers is a distinct area of the basal region of the labellum, situated above and adjacent to the speculum, separating the labellum from the stigmatic cavity [66,68]. In most *Ophrys* species, such as *O. tenthredinifera* and *O. scolopax Cav.*, the basal field is a clearly distinguishable feature of the labellum. However, in species from the section *Pseudophrys*, like *O. lutea* and *O. fusca*, the labellum lacks this differentiated area [59,66,68].

The controversy regarding the presence of a basal field in *O. speculum* arises from varying interpretations of the flower’s structure by different researchers [52,59,64,67]. This issue can only be resolved through a detailed micromorphological comparison of the labellum and stigmatic cavity between *O. speculum* and other *Ophrys* species, aiming to establish homology between the floral areas. Homology is typically assessed based on three criteria: (i) similarity in relative position; (ii) sharing a common special feature; and/or (iii) the presence of intermediate forms [69,70]. In this context, our observations provide two lines of evidence supporting the existence of a basal field in the flowers of *O. speculum*.

Firstly, the flat epidermal cells with a densely ridged surface found in the glabrous floor of the stigmatic cavity in *O. speculum* are similar to those in the floor of the stigmatic cavity in other *Ophrys* species, particularly in its close relatives *O. bombyliflora* and *O. tenthredinifera* but also in *O. scolopax* [52,59]. Additionally, despite being longer, the floor of the stigmatic cavity in *O. speculum* occupies the same relative position as the floor of the stigmatic cavity in those *Ophrys* species, being framed by two pairs of elongated crests or labia, which are also part of the stigmatic cavity. Consequently, we interpret this glabrous area in *O. speculum* as an exceptionally long, narrow floor homologous to the floor of the stigmatic cavity of other *Ophrys* species [52,59], thereby challenging previous interpretations that misidentify this area as a poorly defined basal field in *O. speculum* [64,67].

Secondly, the basal region of the labellum in *O. speculum* also presents an area covered with trichomes, positioned above the speculum and directly below—and contiguous to—the floor of the stigmatic cavity, similar to the basal labellum region in other *Ophrys* species [52,54,59,64]. In *O. speculum*, this area of trichomes is confined to the cup-shaped concavity located between the distal ends of the internal labia of the stigmatic cavity and it is enclosed by an augmented portion of the labellum. Despite its unique location, this circumscribed area of the labellum of *O. speculum* presents the same relative position and shares the same type of epidermal cells (trichomes) as the basal field of other *Ophrys* species, although these trichomes vary in height and shape across species [52,59]. Meeting the first two criteria for homology [69,70], we categorise this distinctive cupuliform concavity as a definitive basal field. Supporting our interpretation are observations of collapsed trichomes in early buds of *O. speculum* subsp. *lusitanica*, the subspecies with the narrowest floor and most pronounced internal labia. This phenomenon likely results from the trichomes being compactly packed into the limited space between the enlarged labia of the stigmatic cavity, and thereby subjected to significant compression. This suggests that cell collapse may be a way to create additional space necessary for the complete development of typical basal field trichomes. Further ultrastructural studies are recommended to clarify the cause of this cellular collapse.

Although the identification of a distinct circumscribed area—corresponding to the cupuliform concavity detailed here—in the basal region of the labellum of *O. speculum* has been well documented [47,60,64,66,67,68], this study is the first to demonstrate that this specific area represents the basal field of the species.

In conclusion, based on its relative position within the flower and its micromorphological characteristics, we suggest that the cupuliform concavity of *O. speculum*, provided with sub-conical to attenuate trichomes, constitutes a well-defined basal field, and this area is homologous to the basal fields found in the labella of other *Ophrys* species [52,59], rather than being a structure exclusive to the *O. speculum* group [64]. However, the unique location and distinct characteristics of the basal field of *O. speculum* appear to be unparalleled within the genus *Ophrys*.

The significance of discussing whether *O. speculum* displays a clear basal field in the labellum extends beyond mere theoretical considerations. Indeed, analysing the floral structure of *O. speculum* has enabled the identification of homologies between floral parts across the genus *Ophrys*, forming the basis of the character definition necessary for conducting morphological phylogenetic analyses [71,72]. In effect, the micromorphological and anatomical data gathered on *O. speculum* in this study has previously been combined with equivalent data from representative species of the other three closely related species groups and used for aiding in reconstructing the phylogenetic relationships and the ancestral states of floral characters within this clade [59].

### 3.2. Micromorphology and Pollination

#### 3.2.1. Morphologically Distinct Epidermal Cell Types

Here, we show precise micromorphological data on the labellum and stigmatic cavity of *O. speculum*, which can be accurately compared with similar data from closely related species and used in phylogenetic analyses of the genus *Ophrys* [58,59].

This study reveals that the adaxial surface of the labellum and stigmatic cavity of *O. speculum* presents seven different morphologically distinct epidermal cell types, closely matching the cell diversity found in *O. tenthredinifera* and *O. scolopax*, which both exhibit eight different types. This contrasts with the 11 cell types displayed by *O. bombyliflora* and the no more than three cell types found in *O. fusca*, *O. lutea*, and other species from section *Pseudophrys* [52,54,59,64]. Revisiting our criteria for defining morphologically distinct epidermal cell types [73], we revised our previous assumption that *O. fusca* and *O. lutea* had four different types [54]; we now recognise only three cell types. This adjustment is necessary since the long attenuated trichomes with enlarged bases found in the basal region of the labellum are actually rather similar to those trichomes covering the apical region, previously misidentified as acuminate trichomes by Ascensão et al. [54]. These trichomes, whether straight or sinuate, do not represent a fourth cell type. Contrasting with the lower diversity reported by Bradshaw et al. [64], who noted only four cell types in *O. speculum*—misinterpreting a single cell type as occurring across the speculum, pseudoeyes, and the floor of the stigmatic cavity, erroneously considered as a basal field by the same authors—our findings highlight that each of these areas has a morphologically distinct epidermal cell type, with only the pseudoeyes presenting smooth, flat lenticular cells.

The speculum of the flowers examined in our study consists of flat to lenticular cells with very fine parallel-arranged cuticular striations, a finding that is somewhat inconsistent with what was described by Bradshaw et al. [64] and Vignolini et al. [65]. Although comparable striations are evident on the surface of the speculum cells of *O. speculum* subsp. *lusitanica* in their scanning and transmission electron micrographs, Bradshaw et al. [64] considered these cells as totally smooth. Likewise, in their subsequent investigation focused on the speculum of *O. speculum*, the authors confirmed the extremely flat and smooth surface of these cells [65]. The apparent discrepancy between our findings and those reported by Bradshaw et al. [64] and particularly by Vignolini et al. [65] may be attributable to the different provenance of the flowers examined in the three studies, namely flowers from naturally occurring plants in our study versus flowers predominantly [64] or exclusively [65] collected from seed-grown plants in greenhouse conditions. Edaphoclimatic factors such as soil moisture, light intensity, air temperature, and humidity vary significantly between wild and cultivated environments and could influence the process of cuticular wax deposition and, consequently, cuticle micromorphology [74]. Furthermore, this discrepancy may also stem from the analysis of flowers at different developmental stages across the different studies. Additionally, the sample size examined so far may not fully capture the intraspecific natural variability.

Regardless of whether the speculum cells of *O. speculum* are completely smooth or finely ridged, it is clear that these cells create a highly reflective, mirror-like surface, which is a primary factor in the glossiness and intense specular reflection of light observed in the speculum area of this species [65]. The thin film we detected in the basal region of the speculum could be associated with the mirror-coating effect of its surface, as described by Vignolini et al. [65]. This film may represent the thin epicuticular wax film that virtually occurs on all plant surfaces covered with a cuticle, although it is often challenging to detect by scanning electron microscopy [73,75,76,77]. Additional histochemical tests are recommended to substantiate this hypothesis. Furthermore, the irregular cuticular projections noted in an early bud at the portion of the speculum that undergoes more drastic distension during labellum expansion, prior to flower anthesis, are expected to stretch and disappear, resulting in a nearly smooth cuticular coating over the speculum in open flowers. Similar changes in the cuticle morphology were observed during the development of grape berries [78].

The presence of flat lenticular cells in the speculum is shared with the closely related *O. bombyliflora* and *O. tenthredinifera* [52,64], yet the parallel arrangement of the fine cuticular striations in *O. speculum* distinguishes it within the genus, contrasting with the typical spiralled cuticular pattern seen in the speculum’s cells in other *Ophrys* species [52,54,62,64]. Apart from *O. speculum*, only a few species within the group of *O. bertolonii* Moretti, which are pollinated by *Chalicodoma* bees [68], were found to exhibit exclusively flat epidermal cells in the speculum [64]. Additionally, the speculum’s epidermis in *O. speculum* predominantly exhibits cells with a hexagonal outline, occasionally mixed with cells with a pentagonal or a heptagonal outline, a pattern analogous to that of the exoskeleton of an iridescent beetle [79]. The inclusion of topological defects (five- and seven-sided polygons) in a hexagonal lattice, necessary to outline a curved surface [80,81], may explain the cell arrangement in the speculum area of *O. speculum*. This area covers the majority of the convex central labellum lobe, which is itself a notably curved surface.

#### 3.2.2. Visual and Tactile Mimicry

From our detailed micromorphological comparative study, we propose new evidence of mimicry between the basal labellum region of the *O. speculum* flower and the thorax and wings of the female of its wasp pollinator, *Dasyscolia ciliata*, specifically between the following: (i) the oblong to shield-shaped basal field within the cupuliform concavity of the flower and the hairy triangular area corresponding to the pilosity of the scutellum and metanotum, on the wasp’s thorax, not covered by the crossed forewings; (ii) the two pairs of labia of the stigmatic cavity of the flower and the prominent costal veins in the proximal area of the crossed wings; (iii) the glabrous floor of the stigmatic cavity and the shiny scutum of the wasp’s thorax; (iv) the series of labellar grooves and crests and the veins of the median area of the wings; (v) the orderly rows of cuticular depositions parallel to the apical margin of the labellum and the rippled surface of the distal area of the wings; and (vi) the heterogeneous cuticular pattern in the lateral labellum lobes and the cuticular wrinkles on the dorsal surface of the wings.

Moreover, our findings align with other previously recognised similarities between the flower of *O. speculum* and the female wasp. For instance, we concur with Devillers and Devillers-Terschuren [66] and Paulus [43] that the flower’s pseudoeyes mimic the wasp’s tegulae, although they are positioned higher in the flower. The submarginal band of contorted trichomes around the labellum resembles the brownish-orange-coloured hairiness of the legs and the abdomen of the female wasp, the latter being composed of slightly spiralled hairs, as also described by Ågren et al. [60]. Notably, Paulus [43] noted that this colouration match is found only between *O. speculum* subsp. *speculum* and *D. ciliata* subsp. *ciliata*, the wasp subspecies that is predominantly distributed over the western Mediterranean region [82]. The other subspecies of this wasp, i.e., *D. ciliata* subsp. *araratica* (Radoskovsky, 1890), which occurs only in the eastern Mediterranean region [82], exhibits a darker colouration that is reminiscent of the darker-coloured flowers of an eastern population of this orchid, *O. speculum* Link var. *orientalis* (Paulus) Kreutz [43].

Both the submarginal hairiness and the unique configuration of the stigmatic cavity and basal labellum region of *O. speculum* should provide tactile stimuli to male wasps, similarly to those offered by the hairiness of the female, hence guiding males to the correct position upon the labellum so that effective pollination could take place [11,37,41,43,60]. The importance of tactile cues for the success of pseudocopulation in *Ophrys* was also inferred from certain micromorphological details on the labellum of other species [42,52,54,60] and has long been tested in the field through choice experiments with their respective male insect pollinators [11,37,46,83].

Our observations support the widely accepted theory that the speculum of this *Ophrys* flower mimics the crossed wings of a virgin female of the *D. ciliata* wasp on the ground [11,41,43,60]. Specifically, the outline of the apical region of the speculum is quite similar to the small, inverted V formed by the partial overlapping of the wasp’ forewings, a feature also described by Ågren et al. [60]. Remarkably, the wings of this wasp exhibit a nearly hairless, rippled dorsal surface [47,60], similar to those of another scoliid wasp [84]. In contrast, the dorsal wings’ surface from females of other *Ophrys* pollinators is provided with short hairs that are either uniformly distributed along the veins and the wing membrane, as seen in *Argogorytes* wasps and *Andrena* bees, or densely packed on the veins, as in *Eucera* bees [47,60]. The fact that the wings’ surface of a female *D. ciliata* wasp is nearly hairless may thus explain why the speculum of *O. speculum* is entirely composed of flat to slightly convex cells, which contrast with the papillae and/or short trichomes with flattened bases typically found in the speculum of *Ophrys* species pollinated by males of those insect genera [52,54,59,62,64]. In addition to mimicking the shape, blue reflection, and glabrous surface of the female wasp’s crossed wings, the speculum of *O. speculum* also mimics the strong UV reflectance typical of the wasps’ wings, which was found to be a highly attractive visual signal for male hymenopterans [11,33,43,65,85]. Consequently, the speculum constitutes a secondary key visual stimulus that, in combination with the primary key odour signal, seems to play a decisive role in the short-range attraction of male insect pollinators to the flower [11,43]. Further field experiments with diverse *Ophrys* species and their respective insect pollinators are yet needed to demonstrate which labellar features act effectively as visual stimuli for pollinators [30,32,36,37,39].

Among the two investigated subspecies of *O. speculum*, only *O. speculum* subsp. *speculum*, which is widespread around the Mediterranean Basin, is consistently pollinated by male *D. ciliata* wasps [11,20,86,87]. Conversely, for *O. speculum* subsp. *lusitanica*, confined to the southwest of the Iberian Peninsula [67,68,86], the specific pollinator remains unidentified due to limited pollination records [88]. The practically similar flower structure and micromorphology observed between these two subspecies, along with their strong resemblance to the female *D. ciliata* wasp, suggest that males of this wasp may be potential pollinators of *O. speculum* subsp. *lusitanica*. Nonetheless, this hypothesis can only be tested after analysing the floral scent of this subspecies and conducting behavioural field tests with males of that particular wasp, given the primary role of the scent in attracting pollinators [24]. Furthermore, a third taxon, *O. speculum* Link subsp. *regis-ferdinandii* Acht. & Kellerer ex Kuzmanov (synonym: *O. regis-ferdinandii* (Renz) Buttler), is closely related to the two studied taxa, hence forming the so-called ‘*O. speculum* group’ recognised by several authors [39,56,58,66,68], but its distribution is restricted to the eastern Aegean islands and neighbouring parts of Anatolia [20,68,86]. While the flower morphology of *O. speculum* subsp. *regis-ferdinandii* appears similar to the other two subspecies [39,64], its potential pollinator might be a male of the distantly related bee-like hoverfly *Merodon velox* (Diptera: Syrphidae) [43].

### 3.3. The Significance of the Osmophore in Ophrys speculum

#### 3.3.1. Osmophore Location, Structure, and Secretion

This study provides evidence for the presence of an osmophore in the apical region of the labellum of *O. speculum*. The characteristics exhibited by the dome-shaped papillae and the underlying two-to-three layers of parenchyma cells at the apical margin of the labellum are comparable to most features representative of an osmophore [53,89,90,91,92,93]. These papillae present typical features of secretory cells [94] and are also similar to the glandular cells occurring in the labellum margin and/or in the apical appendix of other *Ophrys* species with an osmophore [52,54,59], including *O. bombyliflora*, *O. tenthredinifera*, and species in the section *Pseudophrys* such as *O. fusca* and *O. lutea*, which together form a clade with *O. speculum* in molecular phylogenetic analyses [55,56,57,58]. The osmophore in *O. speculum* appears to be restricted to the area of the glabrous apical margin of the labellum nearest the margin itself, occupying both the abaxial and the adaxial surfaces of the labellum. In contrast, in *O. fusca* and *O. lutea*, two species that share the absence of an apical appendix in the labellum with *O. speculum*, the osmophore extends to the entire labellum margin and to the abaxial surface of the apical labellar region [54], whereas in the other three osmophore-containing *Ophrys* species, this glandular structure occurs in the apical appendix and also occupies the apical labellum margin in two of them [52,59]. The location of the osmophores of *O. speculum* and other *Ophrys* species in the apical region of the labellum agrees with the most common location of these glands in the flower, i.e., the tips, margins, or prominent areas of sepals, petals, or labella [90,92,93,95].

Our finding that a low amount of a lipophilic secretion with terpenoid compounds was detected in the osmophore cells of *O. speculum* is consistent with the chemical analyses of the flower scent previously performed for this species, which revealed several monoterpenes, sesquiterpenes (primarily cyclosativene), and other terpenoids among the odour compounds [38,47,96]. The detection of such a secretion in the osmophore of *O. speculum* suggests that this glandular structure is likely the site of biosynthesis and release of part of the highly volatile fraction of the odour bouquet, in accordance with what was also proposed for other *Ophrys* species [52]. Although morpho-anatomical and histochemical evidence has been provided for the occurrence of an osmophore in the labellum of seven *Ophrys* taxa ([52,54,59], present study), the functional role of this secretory structure must be confirmed by analysing the spatial distribution of scent emissions in the labellum, including scent chemical composition, ultrastructure of the secretory cells, and gene expression. Previous studies have demonstrated the involvement of osmophore tissues in scent emission in other species with osmophores [91,97,98].

However, unlike the osmophores in other *Ophrys* species, only a minimal starch content was observed in the osmophore of *O. speculum*, specifically in two to three layers of parenchyma cells beneath the glandular epidermis. Its location is similar to that found in *O. bombyliflora*, *O. tenthredinifera,* and *O. scolopax* [52,59] but contrasts to that of *O. fusca* and *O. lutea*, which exhibit abundant starch-rich plastids in the secretory epidermal cells [53,54]. Similarly, no starch was detected in the osmophores of some other terrestrial orchids, which instead contain lipid inclusions in the cytoplasm [53,95,99,100]. Conversely, most species with osmophores exhibit a substantial amount of starch in the subsecretory tissues (e.g., [89,91]). Starch and lipid reserves within osmophore tissues are believed to be important sources of energy and/or precursor molecules for the intense and continuous biosynthesis of the volatile organic compounds of the secretion, which are likely to be emitted continuously from the secretory epidermal cells due to their high volatility [53,89,100]. The minimal starch content in the osmophore tissues of *O. speculum* may suggest low metabolic activity in this secretory structure. However, the glandular epidermal cells have voluminous, occasionally lobed nuclei, indicating intense gene expression potentially linked to partial progressive endoreplication, a phenomenon recently described in the labellum of *O. speculum*, especially in the adjacent submarginal trichomes [101].

#### 3.3.2. The Role of the Osmophore in Pollination and in an Evolutionary Context

The key semiochemicals used by *O. speculum* for attracting males of its specific *D. ciliata* wasp pollinator are three uncommon short-chained oxygenated carboxylic acids: 9-oxodecanoic acid, 9-hydroxydecanoic acid, and 7-hydroxyoctanoic acid [48]. These compounds are presumably more volatile than the long-chained hydrocarbons typically used by various *Ophrys* species as primary attractants for their *Andrena* or *Colletes* bee pollinators [12,38,49,50]. Indeed, despite the fact that equivalent series of long-chained n-alkenes were also detected in high amounts in the cuticle solvent extracts of the labellum of *O. speculum*, these compounds do not constitute the behaviourally active compounds in this species [48], unlike in *Ophrys* species pollinated by solitary bees from the genera *Andrena* and *Colletes* [26,27,49,50,102,103]. Most of these long-chained cuticular hydrocarbons have low vapour pressure at room temperature and are expected to be effective only upon contact or at close range, like some contact sex pheromones in insects [38,104]. For *Ophrys* species that utilise hydrocarbons as the key semiochemicals, it is likely that more volatile compounds, possibly synthesised in the osmophore, are responsible for attracting male pollinators from greater distances [52]. This hypothesis aligns with findings that a highly volatile monoterpene, linalool, emitted from the labellum of *O. exaltata* Ten., serves as an efficient long-range attractant for its specific pollinator, male *Colletes cunicularius* bees [49]. A comparable phenomenon was found in a Neotropical orchid, *Mormolyca ringens* (Lindl.) Schltr., which uses an alkanol (2-heptanol) as long-range attractant, in complement to the long-chained 9-alkene/alkane series that acts at a shorter distance as a sexual behaviour elicitor in its specific eusocial bee pollinator [22]. We have previously proposed a model of pollinator attraction predicting that two different sources of chemical signals play complementary roles in *Ophrys* pollination: (i) highly volatile organic compounds synthesised in the osmophore, primarily located at the apical region of the labellum, serving as long-range attractants; and (ii) long-chained n-alkenes generally found among the constituents of the cuticular waxes [105], which spread probably over the entire labellum surface, acting mainly at close distances or upon contact [52].

In *O. speculum*, the higher volatility of the key signal provided by the short-chained oxygenated carboxylic acids [48] suggests that these compounds may also serve as medium- or long-range attractants for male *D. ciliata* wasps. However, it remains unclear which epidermal cells produce the three key oxygenated carboxylic acids of *O. speculum*, found in minute amounts in the labellum’s cuticle extracts [38,48]. Due to their low molecular mass, it is plausible that these key semiochemicals could be synthesised in the osmophore and extracted from the cuticle of the epidermal cells located at the apical labellum margin. This hypothesis warrants testing through differential analyses of the scent emitted from the osmophore region and the other areas of the labellum, employing both solvent extraction and headspace sorption sampling techniques [24].

Alternatively, the osmophore of *O. speculum* may serve as an additional source of highly volatile semiochemicals, enriching its diverse odour bouquet and offering evolutionary potential for attracting new pollinators. This secretory structure is particularly significant in an orchid species which maintains a fixed one-to-one relationship with a wasp pollinator species that has evolved an unusual mating signal consisting of a few unique compounds, thereby effectively isolating it from other scoliid wasps and sympatric insect species [43,48]. A pollinator shift in *O. speculum* may thus imply a jump to a different taxonomic group of insect pollinators [42], as seen in the case of *O. speculum* subsp. *regis-ferdinandii* if its suspected hoverfly pollinator [43] is confirmed.

The role of the osmophore of *O. speculum* warrants discussion within an evolutionary framework. Molecular phylogenetic analyses conducted in genus *Ophrys* and subtribe Orchidinae showed consistently that *O. speculum* forms a major clade together with *O. bombyliflora*, *O. tenthredinifera*, and section *Pseudophrys*, although the interspecific relationships in this clade vary among analyses [55,56,57,58,106,107]. The two most likely trees derived from the latest morphological phylogenetic analysis for this clade suggest that *O. bombyliflora* and *O. tenthredinifera* are not sister groups [59]. This supports the topology of molecular trees based not only on nuclear ITS data, either alone or in combination with plastid and/or mitochondrial datasets [8,56,106,107], but also on the RAD sequencing data [58] and rejects both the previous morphological cladistic hypothesis [66] and the molecular phylogenetic trees based solely on plastid data [55,56,107].

Additionally, the findings by Francisco et al. [59] align relatively well with the latest molecular phylogenetic analyses in genus *Ophrys* [57,58]. According to the phylogenetic trees obtained by Breitkopf et al. [57] and Bateman et al. [58], *O. speculum* appears as an early-diverging lineage at the base of the clade, forming a sister group to the other three species groups, which have diverged according to the branching order *O. bombyliflora*—*O. tenthredinifera*—section *Pseudophrys*, all nodes receiving strong support level. This topology represents exactly the reverse branching sequence of one of the two most likely trees found by Francisco et al. [59] but is relatively congruent with the topology of the other most likely tree from the same study, which recovered two associations (*O. speculum*–*O. bombyliflora* and *O. tenthredinifera*–section *Pseudophrys*) whose divergence time was impossible to infer by these authors [59]. The phylogenetic analysis by Breitkopf et al. [57], however, estimated that the clade of *O. speculum* had diverged early in the genus, following the split of the earliest diverging lineage of *O. insectifera* L., which was placed with strong support as basally divergent in the genus. Therefore, according to the phylogenetic hypotheses by Breitkopf et al. [57] and Bateman et al. [58], sexual deceptive pollination by wasps seems to be the ancestral state of *Ophrys* orchids, with *O. insectifera* and *O. speculum* emerging as the two earliest branching lineages in the genus, aligning with a previous hypothesis by Kullenberg and Bergström [83].

The diversification of genus *Ophrys* appears to have been driven by ecological opportunities, particularly the availability of new groups of pollinators, the shifts from wasp to bee pollinators being the main promoting factor of species diversification in this orchid genus [57]. Such transitions, progressing from wasps to *Eucera* bees and then to *Andrena* bees and other bee pollinators, may have been facilitated by the presence of n-alkenes in the cuticular waxes spread over the labellum of *Ophrys* flowers [17,57], which were found to be widespread in subtribe Orchidinae and the ancestral state in genus *Ophrys* [108].

Given the prevalence of osmophores in the genus *Ophrys* ([52,53,54,59], present study) and their occurrence in some species within the subtribe Orchidinae [53,99,109], including species of closely related genera to *Ophrys*, such as *Serapias* and *Himantoglossum* [107], it is likely that the occurrence of an osmophore in the labellum represents a plesiomorphic feature that may have already been present in the most recent common ancestor of genus *Ophrys*. In the light of this, we suggest that shifts in pollinators have been facilitated by the production of highly volatile compounds synthesised in the osmophore.

## 4. Materials and Methods

### 4.1. Taxon Sampling

Inflorescences of *O. speculum* Link subsp. *lusitanica* O.Danesch & E.Danesch (synonym: *O. vernixia* Brot.) and *O. speculum* Link subsp. *speculum* (synonym: *O. ciliata* Biv.) were collected in March and April, between 2005 and 2009, from natural populations in Portugal, in the municipalities of Loures (about 25 km north of Lisbon) and Sesimbra (about 40 km south of Lisbon), respectively. The taxonomic classification adopted here follows Aldasoro and Sáez [67]. A voucher specimen of each taxon was deposited in the Herbarium of the University of Lisbon Botanical Garden (LISU) in Portugal, under the accession numbers LISU 231242 and LISU 231246.

Flowers at three developmental stages were examined in the present study: (1) early bud; (2) late bud; and (3) open flower. The dimensions and age associated with each developmental stage per investigated taxon are shown in Table 1.

One female of *Dasyscolia ciliata* subsp. *ciliata wasp* (Fabricius, 1787; Hymenoptera: Scoliidae), which is the reported pollinator of *O. speculum* subsp. *speculum* [11,20,87], was captured in April 2008 in the vicinity of a population of this subspecies in Serra de Sicó (central-littoral region of Portugal) and examined. Observations were verified on three other specimens of females of *Dasyscolia ciliata* subsp. *ciliata* deposited in Museum collections, namely two specimens deposited in the National Museum of Natural History and Science of the University of Lisbon (MUHNAC; MB07-033829 and MB07-035499) and one specimen deposited in the Science Museum of the University of Coimbra (MCUC; MCUC, ZOO.0005657) in Portugal.

### 4.2. Scanning Electron Microscopy

Four open flowers and two late buds of each of the two subspecies of *O. speculum* and the female wasp captured in Serra de Sicó were fixed with 2.5% glutaraldehyde in 0.1 M sodium phosphate buffer, at pH 7.2, dehydrated in a graded acetone series, critical-point dried and then mounted, sputter-coated with gold, and observed on a JEOL T220 scanning electron microscope (JEOL Ltd., Tokyo, Japan) at 15 or 20 kV.

Flower morphology was described using the terminology of Devillers and Devillers-Terschuren [66]. Morphologically distinct epidermal cell types were defined considering the features of the cell surface, namely, the curvature of the outer cell wall, the cell outline, and the cuticular sculpture (Table 2), following the terminology of Kay et al. [110] and Koch et al. [73]. The terminology used for describing the insect morphology followed the glossary of the Hymenoptera Anatomy Ontology [111].

### 4.3. Stereomicroscopy

Fresh buds and open flowers of the two subspecies of *O. speculum* and the female wasp individual captured in Serra de Sicó were examined under an Olympus SZH-ILLK stereomicroscope (Olympus Optical Co., Ltd., Tokyo, Japan). For the macroscopic visualisation of the scent-producing areas of the flower, two intact freshly opened flowers of *O. speculum* subsp. *lusitanica* were immersed in 0.01% neutral red for 2–4 h [53], rinsed in tap water, and examined. Images were recorded digitally using an Olympus C-7070 Wide Zoom digital camera (Olympus Imaging Corp., Tokyo, Japan).

The three female wasp specimens from Museum collections were examined under the Leica MS5 and MZ95 stereomicroscopes (Leica Microsystems GmbH, Wetzlar, Germany) and photographed using an AmScope MU500 digital microscope camera (AmScope, Irvine, CA, USA) and the ToupTek ToupView software (v. 3.7.6701).

### 4.4. Light Microscopy

For the anatomical study, pieces of the labellum of both subspecies of *O. speculum* in the aforementioned developmental stages (three flowers at each stage, on average) were fixed as described for SEM, embedded in Leica Historesin, sectioned (2 µm thick) using a Leica RM-2155 microtome (Leica Microsystems, Nussloch, Germany), and stained with toluidine blue *O* with post-staining with dilute Lugol [112] or periodic acid–Schiff (PAS) reagent/toluidine blue *O* [113] for general histology, starch, and total polysaccharides; they were stained with Sudan Black B [114] for the detection of lipids. Appropriate controls were also performed.

Segments of the apical margin of the labellum from flowers of *O. speculum* subsp. *lusitanica*, at all three developmental stages, were also fixed with glutaraldehyde, post-fixed with osmium tetroxide, and embedded in Epon-Araldite resin (Electron Microscopy Sciences, Fort Washington, PA, USA), following the procedure described in Francisco and Ascensão [52]. Semi-thin sections (~0.5 µm thick) were obtained with a Sorvall MT-1 ultramicrotome (Sorvall Inc., Norwalk, CT, USA) and stained with Sudan Black B for lipids [114].

For the histochemical characterisation of the osmophore, transverse and longitudinal hand-cut sections of the glabrous apical margin of the labellum were made in fresh late buds and open flowers of the two subspecies of *O. speculum*. The main classes of compounds generally present in the flower scents were investigated using Sudan Black B [115] and neutral red under UV and blue light [116] for the detection of total lipids, and Nadi reagent [117] was used for the detection of terpenoids. Phenolic compounds were detected by their autofluorescence under UV and blue light [118]. Hand-cut sections of the basal and median labellar regions from fresh buds and open flowers of both subspecies were also made and tested for lipids with Sudan IV [115]. Control reactions were performed simultaneously.

Observations were made with a Leica DM-2500 microscope (Leica Microsystems, Wetzlar, Germany) and images were recorded digitally with a Leica DFC-420 camera (Leica Microsystems, Heerbrugg, Switzerland) and the Leica Application Suite software (ver. 2.8.1). For observations under UV and blue light, a Leitz SM-LUX epifluorescence microscope (Leitz-Wetzlar, Wetzlar, Germany) equipped with an HBO 50 W mercury vapour lamp, filter block A (excitation filter BP 340–380, dichroic mirror 450, and barrier filter LP-430), and filter block I2 (excitation filter BP 450–490, barrier filter LP-515) was used.

## 5. Conclusions

This study provides the first evidence for the presence of an osmophore, a fragrance-producing secretory structure, in the labellum of *O. speculum* subsp. *lusitanica* and *O. speculum* subsp. *speculum*, the two representative subspecies of *O. speculum* in the westernmost area of its geographical range, and it sheds new light on the structure of the uncommon stigmatic cavity and labellum of *O. speculum*. In fact, this study is the first to show micromorphological and anatomical evidence for the presence of a distinct basal field in the labellum of this species—located in a unique cup-shaped concavity—and to demonstrate its homology to the basal field found in the labellum of other *Ophrys* species. Additionally, comparisons between the labellum and the female of its specific wasp pollinator, *Dasyscolia ciliata*, details structural similarities that represent new evidence of visual and tactile mimicry—especially between the basal region of the labellum and the female wasp’s thorax and wings—featuring the importance of visual and tactile cues for the success of pollination by sexual deception in *O. speculum.* Analysing these findings in an evolutionary context enabled us to suggest that the presence of an osmophore in the labellum is a plesiomorphic feature in the *Ophrys* genus and the biosynthesis of highly volatile organic compounds in this secretory structure may have facilitated the shifts in pollinators throughout the evolutionary history of these orchids.

## Figures and Tables

**Table 1:** Dimensions and age associated with the three developmental stages of the flowers of *Ophrys speculum* subsp. *lusitanica* and *Ophrys speculum* subsp. *speculum*.

	Dimensions			Age (Number of Days)		
	(Length × Width; mm)			Before Anthesis/At Anthesis		
	Early Bud ^a,^*	Late Bud ^b,^*	Open Flower ^c,T^	Early Bud ^a,^*	Late Bud ^b,^*	Open Flower ^c,Y^
*Ophrys speculum* subsp. *lusitanica*	5 × 3 to 6 × 4	7 × 4 to 8 × 6	13 (2) × 10 (1)	4 to 6	1 to 3	1 to 10 (6)
*Ophrys speculum* subsp. *speculum*	5 × 3 to 7 × 3	7 × 4 to 9 × 6	13 (2) × 11 (2)	3 to 7	1 to 2	1 to 12 (5)

^a^ Early buds exhibit a yellowish-green to green colouration in the labellum and stigmatic cavity, except for the whitish speculum area and the yellowish (in *O. speculum* subsp. *lusitanica*) to pale brown (in *O. speculum* subsp. *speculum*) submarginal hairiness. ^b^ Late buds exhibit a colouration that is becoming increasingly closer to the typical colouration of open flowers: dark-coloured stigmatic cavity and basal labellum region; deep-blue speculum; light-yellowish-brown (in *O. speculum* subsp. *lusitanica*) or dark-reddish (in *O. speculum* subsp. *speculum*) submarginal hairiness; yellow to greenish-yellow (in *O. speculum* subsp. *lusitanica*) or dark reddish (in *O. speculum* subsp. *speculum*) glabrous labellum margin. ^c^ Open flowers are fully expanded flowers that present a fresh appearance and vivid colours in the labellum and stigmatic cavity. * Sample size (n): *O. speculum* subsp. *lusitanica* n = 24; *O. speculum* subsp. *speculum* n = 44. ^T^ Mean dimensions of the labellum (incl. associated stigmatic cavity); standard deviation is given in brackets; sample size (n): *O. speculum* subsp. *lusitanica* n = 13; *O. speculum* subsp. *speculum* n = 20. ^Y^ The minimum number of days in which the flowers exhibit a fresh appearance is given in brackets; sample size (n): *O. speculum* subsp. *lusitanica* n = 14; *O. speculum* subsp. *speculum* n = 26.

**Table 2:** Morphologically distinct epidermal cell types based on features of the cell surface, found in the adaxial surface of the labellum and the stigmatic cavity in *Ophrys speculum*.

Morphologically Distinct Epidermal Cell Types	Surface Features of the Epidermal Cells		
	Curvature of the Outer Cell Wall	Cell Outline	Cuticular Ornamentation
1	flat cells	elongated, polygonal outline	very dense, parallel cuticular striations
2	flat to lenticular cells	isodiametric, polygonal outline	no cuticular striations (smooth surface)
3	sub-conical to attenuate unicellular trichomes	n/a	fine cuticular striations
4	lenticular cells	isodiametric, polygonal outline	dense cuticular striations
5	long, contorted unicellular trichomes	n/a	fine cuticular striations
6	flat to slightly convex cells	isodiametric, hexagonal (occasionally pentagonal or heptagonal) outline	very fine, parallel cuticular striations
7	dome-shaped papillae	n/a	no cuticular striations (smooth surface)

## Data Availability

The original contributions presented in the study are included in the article. Further inquiries can be directed to the corresponding authors.

## References

[1] van der Pijl L., Dodson C.H. (1966). Orchids Flowers, Their Pollination and Evolution.

[2] Dafni A. (1984). Mimicry and deception in pollination. Annu. Rev. Ecol. Syst..

[3] Renner S.S., Waser N.M., Ollerton J. (2005). Rewardless flowers in the angiosperms and the role of insect cognition in their evolution. Plant-Pollinator Interactions: From Specialization to Generalization.

[4] Schiestl F.P. (2005). On the success of a swindle: Pollination by deception in orchids. Naturwissenschaften.

[5] Jersáková J., Johnson S.D., Kindlmann P. (2006). Mechanisms and evolution of deceptive pollination in orchids. Biol. Rev. Camb. Philos. Soc..

[6] Peter C.I., Johnson S.D. (2013). Generalized food deception: Colour signals and efficient pollen transfer in bee-pollinated species of *Eulophia* (Orchidaceae). Bot. J. Linn. Soc..

[7] Gaskett A.C. (2011). Orchid pollination by sexual deception: Pollinator perspectives. Biol. Rev..

[8] Vereecken N.J., Wilson C.A., Hötling S., Schulz S., Banketov S.A., Mardulyn P. (2012). Pre-adaptations and the evolution of pollination by sexual deception: Cope’s rule of specialization revisited. Proc. R. Soc. B.

[9] Phillips R.D., Scaccabarozzi D., Retter B.A., Hayes C., Brown G.R., Dixon K.W., Peakall R. (2014). Caught in the act: Pollination of sexually deceptive trap-flowers by fungus gnats in *Pterostylis* (Orchidaceae). Ann. Bot..

[10] Pouyanne M. (1917). La fécondation des *Ophrys* par les insectes. Bull. Soc. Hist. Nat. Afr. Nord.

[11] Kullenberg B. (1961). Studies in *Ophrys* pollination. Zool. Bidr. Upps..

[12] Schiestl F.P., Ayasse M., Paulus H.F., Löfstedt C., Hansson B.S., Ibarra F., Francke W. (1999). Orchid pollination by sexual swindle. Nature.

[13] Singer R.B., Flach A., Koehler S., Marsaioli A.J., Amaral M.C.E. (2004). Sexual mimicry in *Mormolyca ringens* (Lindl.) Schltr. (Orchidaceae: Maxillariinae). Ann. Bot..

[14] Ciotek L., Giorgis P., Benitez-Vieyra S., Cocucci A.A. (2006). First confirmed case of pseudocopulation in terrestrial orchids of South America: Pollination of *Geoblasta pennicillata* (Orchidaceae) by *Campsomeris bistrimacula* (Hymenoptera, Scoliidae). Flora.

[15] Peakall R., Ebert D., Poldy J., Barrow R.A., Francke W., Bower C.C., Schiestl F.P. (2010). Pollinator specificity, floral odour chemistry and the phylogeny of Australian sexually deceptive *Chiloglottis* orchids: Implications for pollinator-driven speciation. New Phytol..

[16] Gaskett A.C. (2012). Floral shape mimicry and variation in sexually deceptive orchids with a shared pollinator. Biol. J. Linn. Soc..

[17] Cuervo M., Rakosy D., Martel C., Schulz S., Ayasse M. (2017). Sexual deception in the *Eucera*-pollinated *Ophrys leochroma*: A chemical intermediate between wasp- and *Andrena*-pollinated species. J. Chem. Ecol..

[18] Baguette M., Bertrand J.A.M., Stevens V.M., Schatz B. (2020). Why are there so many bee-orchid species? Adaptive radiation by intra-specific competition for mnesic pollinators. Biol. Rev..

[19] Ayasse M., Paxton R.J., Tengö J. (2001). Mating behavior and chemical communication in the order Hymenoptera. Annu. Rev. Entomol..

[20] Paulus H.F., Gack C. (1990). Pollinators as prepollinating isolation factors: Evolution and speciation in *Ophrys* (Orchidaceae). Isr. J. Bot..

[21] Singer R.B. (2002). The pollination mechanism in *Trigonidium obtusum* Lindl (Orchidaceae: Maxillariinae): Sexual mimicry and trap-flowers. Ann. Bot..

[22] Flach A., Marsaioli A.J., Singer R.B., Amaral M.C.E., Menezes C., Kerr W.E., Batista-Pereira L.G., Corrêa A.G. (2006). Pollination by sexual mimicry in *Mormolyca ringens*: A floral chemistry that remarkably matches the pheromones of virgin queens of *Scaptotrigona* sp.. J. Chem. Ecol..

[23] Schiestl F.P., Schlüter P.M. (2009). Floral isolation, specialized pollination, and pollinator behavior in orchids. Annu. Rev. Entomol..

[24] Perkins J., Hayashi T., Peakall R., Flematti G.R., Bohman B. (2023). The volatile chemistry of orchid pollination. Nat. Prod. Rep..

[25] Scopece G., Musacchio A., Widmer A., Cozzolino S. (2007). Patterns of reproductive isolation in mediterranean deceptive orchids. Evolution.

[26] Xu S., Schlüter P.M., Scopece G., Breitkopf H., Gross K., Cozzolino S., Schiestl F.P. (2011). Floral isolation is the main reproductive barrier among closely related sexually deceptive orchids. Evolution.

[27] Xu S., Schlüter P.M., Grossniklaus U., Schiestl F.P. (2012). The genetic basis of pollinator adaptation in a sexually deceptive orchid. PLoS Genet..

[28] Xu S., Schlüter P.M., Schiestl F.P. (2012). Pollinator-driven speciation in sexually deceptive orchids. Int. J. Ecol..

[29] Peakall R., Whitehead M.R. (2014). Floral odour chemistry defines species boundaries and underpins strong reproductive isolation in sexually deceptive orchids. Ann. Bot..

[30] Spaethe J., Moser W.H., Paulus H.F. (2007). Increase of pollinator attraction by means of a visual signal in the sexually deceptive orchid, *Ophrys heldreichii* (Orchidaceae). Plant Syst. Evol..

[31] Benitez-Vieyra S., Medina A.M., Cocucci A.A. (2009). Variable selection patterns on the labellum shape of *Geoblasta pennicillata*, a sexually deceptive orchid. J. Evol. Biol..

[32] Streinzer M., Paulus H.F., Spaethe J. (2009). Floral colour signal increases short-range detectability of a sexually deceptive orchid to its bee pollinator. J. Exp. Biol..

[33] Gaskett A.C., Herberstein M.E. (2010). Colour mimicry and sexual deception by Tongue orchids (*Cryptostylis*). Naturwissenschaften.

[34] Phillips R.D., Xu T., Hutchinson M.F., Dixon K.W., Peakall R. (2013). Convergent specialization—The sharing of pollinators by sympatric genera of sexually deceptive orchids. J. Ecol..

[35] Sedeek K.E.M., Scopece G., Staedler Y.M., Schönenberger J., Cozzolino S., Schiestl F.P., Schlüter P.M. (2014). Genic rather than genome-wide differences between sexually deceptive *Ophrys* orchids with different pollinators. Mol. Ecol..

[36] Stejskal K., Streinzer M., Dyer A., Paulus H.F., Spaethe J. (2015). Functional significance of labellum pattern variation in a sexually deceptive orchid (*Ophrys heldreichii*): Evidence of individual signature learning effects. PLoS ONE.

[37] Rakosy D., Cuervo M., Paulus H.F., Ayasse M. (2017). Looks matter: Changes in flower form affect pollination effectiveness in a sexually deceptive orchid. J. Evol. Biol..

[38] Ayasse M., Stökl J., Francke W. (2011). Chemical ecology and pollinator-driven speciation in sexually deceptive orchids. Phytochemistry.

[39] Bateman R.M., Rudall P.J. (2023). Morphological continua make poor species: Genus-wide morphometric survey of the European bee orchids (*Ophrys,* L.). Biology.

[40] Darwin C. (1862). On the Various Contrivances by Which BRITISH and Foreign Orchids Are Fertilised by Insects, and on the Good Effects of Intercrossing.

[41] Correvon H., Pouyanne M. (1916). Un curieux cas de mimétisme chez les Ophrydées. J. Soc. Nat. Hort. Fr..

[42] Vereecken N.J., Francisco A., Edens-Meier R., Bernhardt P. (2014). Ophrys pollination: From Darwin to the present day. Darwin’s Orchids: Then and Now.

[43] Paulus H.F. (2006). Deceived males—Pollination biology of the Mediterranean orchid genus *Ophrys* (Orchidaceae). J. Eur. Orchid..

[44] Joffard N., Massol F., Grenié M., Montgelard C., Schatz B. (2019). Effect of pollination strategy, phylogeny and distribution on pollination niches of Euro-Mediterranean orchids. J. Ecol..

[45] Godfery M.J. (1928). Classification of the genus *Ophrys*. J. Bot..

[46] Kullenberg B., Borg-Karlson A.-K., Kullenberg A.-L. (1984). Field studies on the behaviour of the *Eucera nigrilabris* male in the odour flow from flower labellum extract of *Ophrys tenthredinifera*. Nova Acta Regiae Soc. Sci. Ups. Ser. V:C.

[47] Borg-Karlson A.-K. (1990). Chemical and ethological studies of pollination in the genus *Ophrys* (Orchidaceae). Phytochemistry.

[48] Ayasse M., Schiestl F.P., Paulus H.F., Ibarra F., Francke W. (2003). Pollinator attraction in a sexually deceptive orchid by means of unconventional chemicals. Proc. R. Soc. B.

[49] Mant J.G., Brändli C., Vereecken N.J., Schulz C.M., Francke W., Schiestl F.P. (2005). Cuticular hydrocarbons as sex pheromone of *Colletes cunicularius* (Hymenoptera: Colletidae) and the key to its mimicry by the sexually deceptive orchid, *Ophrys exaltata*. J. Chem. Ecol..

[50] Stökl J., Twele R., Erdmann D.H., Francke W., Ayasse M. (2008). Comparison of the flower scent of the sexually deceptive orchid *Ophrys iricolor* and the female sex pheromone of its pollinator *Andrena morio*. Chemoecology.

[51] Gögler J., Twele R., Francke W., Ayasse M. (2011). Two phylogenetically distinct species of sexually deceptive orchids mimic the sex pheromone of their single common pollinator, the cuckoo bumblebee *Bombus vestalis*. Chemoecology.

[52] Francisco A., Ascensão L. (2013). Structure of the osmophore and labellum micromorphology in the sexually deceptive orchids *Ophrys bombyliflora* and *Ophrys tenthredinifera* (Orchidaceae). Int. J. Plant Sci..

[53] Vogel S. (1990). The Role of Scent Glands in Pollination: On the Structure and Function of Osmophores.

[54] Ascensão L., Francisco A., Cotrim H., Pais M.S. (2005). Comparative structure of the labellum in *Ophrys fusca* and *O. lutea* (Orchidaceae). Am. J. Bot..

[55] Soliva M., Kocyan A., Widmer A. (2001). Molecular phylogenetics of the sexually deceptive orchid genus *Ophrys* (Orchidaceae) based on nuclear and chloroplast DNA sequences. Mol. Phylogenet. Evol..

[56] Devey D.S., Bateman R.M., Fay M.F., Hawkins J.A. (2008). Friends or relatives? Phylogenetics and species delimitation in the controversial European orchid genus *Ophrys*. Ann. Bot..

[57] Breitkopf H., Onstein R.E., Cafasso D., Schlüter P.M., Cozzolino S. (2015). Multiple shifts to different pollinators fuelled rapid diversification in sexually deceptive *Ophrys* orchids. New Phytol..

[58] Bateman R.M., Sramkó G., Paun O. (2018). Integrating restriction site-associated DNA sequencing (RAD-seq) with morphological cladistic analysis clarifies evolutionary relationships among major species groups of bee orchids. Ann. Bot..

[59] Francisco A., Porto M., Ascensão L. (2015). Morphological phylogenetic analysis of *Ophrys* (Orchidaceae): Insights from morpho-anatomical floral traits into the interspecific relationships in an unresolved clade. Bot. J. Linn. Soc..

[60] Ågren L., Kullenberg B., Sensenbaugh T. (1984). Congruences in pilosity between three species of *Ophrys* (Orchidaceae) and their hymenopteran pollinators. Nova Acta Regiae Soc. Sci. Ups. Ser. V:C.

[61] Borg-Karlson A.-K., Groth I., Ägren L., Kullenberg B. (1993). Form-specific fragrances from *Ophrys insectifera* L. (Orchidaceae) attract species of different pollinator genera. Evidence of sympatric speciation?. Chemoecology.

[62] Servettaz O., Bino Maleci L., Grünanger P. (1994). Labellum micromorphology in the *Ophrys bertolinii* agg. and some related taxa (Orchidaceae). Plant Syst. Evol..

[63] Cortis P., Vereecken N.J., Schiestl F.P., Lumaga M.R.B., Scrugli A., Cozzolino S. (2009). Pollinator convergence and the nature of species’ boundaries in sympatric Sardinian *Ophrys* (Orchidaceae). Ann. Bot..

[64] Bradshaw E., Rudall P.J., Devey D.S., Thomas M.M., Glover B.J., Bateman R.M. (2010). Comparative labellum micromorphology of the sexually deceptive temperate orchid genus *Ophrys*: Diverse epidermal cell types and multiple origins of structural colour. Bot. J. Linn. Soc..

[65] Vignolini S., Davey M.P., Bateman R.M., Rudall P.J., Moyroud E., Tratt J., Malmgren S., Steiner U., Glover B.J. (2012). The mirror crack’d: Both pigment and structure contribute to the glossy blue appearance of the mirror orchid, *Ophrys speculum*. New Phytol..

[66] Devillers P., Devillers-Terschuren J. (1994). Essai d’analyse systématique du genre *Ophrys*. Nat. Belg..

[67] Aldasoro J.J., Sáez L., Aedo C., Herrero A. (2005). *Ophrys*, L.. Flora Iberica: Plantas Vasculares de la Península Ibérica e Islas Baleares.

[68] Delforge P. (2005). Guide des Orchidées d’Europe, d’Afrique du Nord et du Proche-Orient.

[69] Sattler R., Hall B.K. (1994). Homology, homeosis, and process morphology in plants. Homology: The Hierarchical Basis of Comparative Biology.

[70] Chin S.-w., Lutz S., Wen J., Potter D. (2013). The bitter and the sweet: Inference of homology and evolution of leaf glands in *Prunus* (Rosaceae) through anatomy, micromorphology, and ancestral–character state reconstruction. Int. J. Plant Sci..

[71] Kearney M., Rieppel O. (2006). Rejecting “the given” in systematics. Cladistics.

[72] Nixon K.C., Carpenter J.M. (2012). On homology. Cladistics.

[73] Koch K., Bhushan B., Barthlott W. (2008). Diversity of structure, morphology and wetting of plant surfaces. Soft Matter.

[74] Koch K., Hartmann K.D., Schreiber L., Barthlott W., Neinhuis C. (2006). Influences of air humidity during the cultivation of plants on wax chemical composition, morphology and leaf surface wettability. Env. Environ. Exp. Bot..

[75] Barthlott W., Neinhuis C., Cutler D., Ditsch F., Meusel I., Theisen I., Wilhelmi H. (1998). Classification and terminology of plant epicuticular waxes. Bot. J. Linn. Soc..

[76] Jetter R., Schäffer S., Riederer M. (2000). Leaf cuticular waxes are arranged in chemically and mechanically distinct layers: Evidence from *Prunus laurocerasus* L.. Plant Cell Environ..

[77] Jeffree C.E., Riederer M., Müller C. (2006). The fine structure of the plant cuticle. Biology of the Plant Cuticle.

[78] Casado C.G., Heredia A. (2001). Ultrastructure of the cuticle during growth of the grape berry (*Vitis vinifera*). Physiol. Plant..

[79] Sharma V., Crne M., Park J.O., Srinivasarao M. (2009). Structural origin of circularly polarized iridescence in jeweled beetles. Science.

[80] Bausch A.R., Bowick M.J., Cacciuto A., Dinsmore A.D., Hsu M.F., Nelson D.R., Nikolaides M.G., Travesset A., Weitz D.A. (2003). Grain boundary scars and spherical crystallography. Science.

[81] Vitelli V., Lucks J.B., Nelson D.R. (2006). Crystallography on curved surfaces. Proc. Natl. Acad. Sci. USA.

[82] Osten T., Mitroiu M.-D. (2004). Fauna Europaea: *Dasyscolia ciliata* (Fabricius, 1787). Fauna Europaea, Hymenoptera—Apocrita (excl. Ichneumonoidea).

[83] Kullenberg B., Bergström G. (1976). Hymenoptera Aculeata males as pollinators of *Ophrys* orchids. Zool. Scr..

[84] Sarrazin M., Vigneron J.P., Welch V., Rassart M. (2008). Nanomorphology of the blue iridescent wings of a giant tropical wasp *Megascolia procer javanensis* (Hymenoptera). Phys. Rev. E.

[85] Gaskett A.C., Edens-Meier R., Bernhardt P. (2014). Color and sexual deception in orchids: Progress toward understanding the functions and pollinator perception of floral color. Darwin’s Orchids: Then and Now.

[86] Pedersen H.Æ., Faurholdt N. (2007). Ophrys, the Bee Orchids of Europe.

[87] Lara Ruiz J. (2023). Algunos polinizadores potenciales de *Ophrys,* L. 1753 (Orchidaceae) detectados en la sierra de Alcaraz (Albacete, SE península ibérica). Sabuco.

[88] Lara Ruiz J. (2010). Polinizadores y visitantes de *Ophrys* L. en la Península Ibérica e Islas Baleares. Micobotánica-Jaén Año V.

[89] Pridgeon A.M., Stern W.L. (1983). Ultrastructure of osmophores in *Restrepia* (Orchidaceae). Am. J. Bot..

[90] Pridgeon A.M., Stern W.L. (1985). Osmophores of *Scaphosepalum* (Orchidaceae). Bot. Gaz..

[91] Vogel S., Hadacek F. (2004). Contributions to the functional anatomy and biology of *Nelumbo nucifera* (Nelumbonaceae) III. An ecological reappraisal of floral organs. Plant Syst. Evol..

[92] Pansarin L.M., Pansarin E.R., Sazima M. (2014). Osmophore structure and phylogeny of *Cirrhaea* (Orchidaceae, Stanhopeinae). Bot. J. Linn. Soc..

[93] Possobom C.C.F., Guimarães E., Machado S.R. (2015). Structure and secretion mechanisms of floral glands in *Diplopterys pubipetala* (Malpighiaceae), a neotropical species. Flora.

[94] Fahn A. (1988). Secretory tissues in vascular plants. New Phytol..

[95] Sanguinetti A., Buzatto C.R., Pedron M., Davies K.L., Ferreira P.M.A., Maldonado S., Singer R.B. (2012). Floral features, pollination biology and breeding system of *Chloraea membranacea* Lindl. (Orchidaceae: Chloraeinae). Ann. Bot..

[96] El-Sayed A.M. The Pherobase: Database of Pheromones and Semiochemicals. Floral Volatiles of *Ophrys speculum*. http://www.pherobase.com/database/floral/floral-taxa-species-Ophrys-speculum.php.

[97] Skubatz H., Kunkel D.D., Howald W.N., Trenkle R., Mookherjee B. (1996). The *Sauromatum guttatum* appendix as an osmophore: Excretory pathways, composition of volatiles and attractiveness to insects. New Phytol..

[98] van der Niet T., Hansen D.M., Johnson S.D. (2011). Carrion mimicry in a South African orchid: Flowers attract a narrow subset of the fly assemblage on animal carcasses. Ann. Bot..

[99] Stpiczyńska M. (2001). Osmophores of the fragrant orchid *Gymnadenia conopsea* L. (Orchidaceae). Acta Soc. Bot. Pol..

[100] Wiemer A.P., Moré M., Benitez-Vieyra S., Cocucci A.A., Raguso R.A., Sérsic A.N. (2009). A simple floral fragrance and unusual osmophore structure in *Cyclopogon elatus* (Orchidaceae). Plant Biol..

[101] Bateman R.M., Guy J.J., Rudall P.J., Leitch I.J., Pellicer J., Leitch A.R. (2018). Evolutionary and functional potential of ploidy increase within individual plants: Somatic ploidy mapping of the complex labellum of sexually deceptive bee orchids. Ann. Bot..

[102] Stökl J., Schlüter P.M., Stuessy T.F., Paulus H.F., Fraberger R., Erdmann D., Schulz C., Francke W., Assum G., Ayasse M. (2009). Speciation in sexually deceptive orchids: Pollinator-driven selection maintains discrete odour phenotypes in hybridizing species. Biol. J. Linn. Soc..

[103] Vereecken N.J., Cozzolino S., Schiestl F.P. (2010). Hybrid floral scent novelty drives pollinator shift in sexually deceptive orchids. BMC Evol. Biol..

[104] Mori K., Herrmann A. (2010). Pheromones in chemical communication. The Chemistry and Biology of Volatiles.

[105] Buschhaus C., Jetter R. (2011). Composition differences between epicuticular and intracuticular wax substructures: How do plants seal their epidermal surfaces?. J. Exp. Bot..

[106] Bateman R.M., Pridgeon A.M., Chase M.W. (1997). Phylogenetics of subtribe Orchidinae (Orchidoideae, Orchidaceae) based on nuclear ITS sequences. 2. Infrageneric relationships and reclassification to achieve monophyly of *Orchis sensu stricto*. Lindleyana.

[107] Inda L.A., Pimentel M., Chase M.W. (2012). Phylogenetics of tribe Orchideae (Orchidaceae: Orchidoideae) based on combined DNA matrices: Inferences regarding timing of diversification and evolution of pollination syndromes. Ann. Bot..

[108] Schiestl F.P., Cozzolino S. (2008). Evolution of sexual mimicry in the orchid subtribe Orchidinae: The role of preadaptations in the attraction of male bees as pollinators. BMC Evol. Biol..

[109] Barone Lumaga M.R., Pellegrino G., Bellusci F., Perrotta E., Perrotta I., Musacchio A. (2012). Comparative floral micromorphology in four sympatric species of *Serapias* (Orchidaceae). Bot. J. Linn. Soc..

[110] Kay Q.O.N., Daoud H.S., Stirton C.H. (1981). Pigment distribution, light reflection and cell structure in petals. Bot. J. Linn. Soc..

[111] Hymenoptera Anatomy Consortium The Hymenoptera Glossary. http://glossary.hymao.org.

[112] Gutmann M. (1995). Improved staining procedures for photographic documentation of phenolic deposits in semithin sections of plant tissue. J. Microsc..

[113] Feder N., O’Brien T.P. (1968). Plant microtechnique: Some principles and new methods. Am. J. Bot..

[114] Parham R.A., Kaustinen H.M. (1976). Differential staining of tannin in sections of epoxy-embedded plant cells. Stain Technol..

[115] Pearse A.G.E. (1985). Histochemistry: Theoretical and Applied. Volume 2, Analytical Technology.

[116] Kirk P.W. (1970). Neutral red as a lipid fluorochrome. Stain Technol..

[117] David R., Carde J.P. (1964). Coloration diférentielle des inclusions lipidiques et terpeniques des pseudophylles du *Pin maritime* au moyen du reactif Nadi. C. R. Acad. Sci..

[118] Harborne J.B. (1998). Phytochemical Methods: A Guide to Modern Techniques of Plant Analysis.

